# Loss of DNA repair mechanisms in cardiac myocytes induce dilated cardiomyopathy

**DOI:** 10.1111/acel.13782

**Published:** 2023-02-03

**Authors:** Chathurika Henpita, Rajesh Vyas, Chastity L. Healy, Tra L. Kieu, Aditi U. Gurkar, Matthew J. Yousefzadeh, Yuxiang Cui, Aiping Lu, Luise A. Angelini, Ryan D. O'Kelly, Sara J. McGowan, Sanjay Chandrasekhar, Rebecca R. Vanderpool, Danielle Hennessy‐Wack, Mark A. Ross, Timothy N. Bachman, Charles McTiernan, Smitha P. S. Pillai, Warren Ladiges, Mitra Lavasani, Johnny Huard, Donna Beer‐Stolz, Claudette M. St. Croix, Simon C. Watkins, Paul D. Robbins, Ana L. Mora, Eric E. Kelley, Yinsheng Wang, Timothy D. O'Connell, Laura J. Niedernhofer

**Affiliations:** ^1^ Department of Biochemistry, Molecular Biology and Biophysics, Institute on the Biology of Aging and Metabolism University of Minnesota Minneapolis Minnesota USA; ^2^ Department of Molecular Medicine Scripps Research Institute Jupiter Florida USA; ^3^ Department of Integrative Biology and Physiology University of Minnesota Minneapolis Minnesota USA; ^4^ Division of Geriatric Medicine, Aging Institute University of Pittsburgh Pittsburgh Pennsylvania USA; ^5^ Department of Chemistry University of California, Riverside Riverside California USA; ^6^ Department of Orthopedic Surgery University of Pittsburgh Pittsburgh Pennsylvania USA; ^7^ Steadman Philippon Research Institute Vail Colorado USA; ^8^ Division of Cardiology, Heart and Vascular Institute University of Pittsburgh Pittsburgh Pennsylvania USA; ^9^ Center for Biologic Imaging University of Pittsburgh Pittsburgh Pennsylvania USA; ^10^ Division of Pulmonary, Allergy, and Critical Care Medicine University of Pittsburgh Pittsburgh Pennsylvania USA; ^11^ Fred Hutchinson Cancer Research Center Seattle Washington USA; ^12^ Department of Comparative Medicine University of Washington Seattle Washington USA; ^13^ Department of Physical Medicine and Rehabilitation Northwestern University and Shirley Ryan Ability Lab Chicago Illinois USA; ^14^ Department of Cell Biology University of Pittsburgh Pittsburgh Pennsylvania USA; ^15^ Division of Pulmonary, Critical Care and Sleep Medicine, College of Medicine The Ohio State University Columbus Ohio USA; ^16^ Department of Physiology and Pharmacology West Virginia University Morgantown West Virginia USA

**Keywords:** cardiomyopathy, congestive heart failure, genotoxic stress, oxidative stress

## Abstract

Cardiomyopathy is a progressive disease of the myocardium leading to impaired contractility. Genotoxic cancer therapies are known to be potent drivers of cardiomyopathy, whereas causes of spontaneous disease remain unclear. To test the hypothesis that endogenous genotoxic stress contributes to cardiomyopathy, we deleted the DNA repair gene *Ercc1* specifically in striated muscle using a floxed allele of *Ercc1* and mice expressing Cre under control of the muscle‐specific creatinine kinase (*Ckmm*) promoter or depleted systemically (*Ercc1* ^−/D^ mice). *Ckmm‐Cre* ^*+/−*^ *;Ercc1* ^*−/fl*^ mice expired suddenly of heart disease by 7 months of age. As young adults, the hearts of *Ckmm‐Cre* ^*+/−*^ *;Ercc1* ^*−/fl*^ mice were structurally and functionally normal, but by 6‐months‐of‐age, there was significant ventricular dilation, wall thinning, interstitial fibrosis, and systolic dysfunction indicative of dilated cardiomyopathy. Cardiac tissue from the tissue‐specific or systemic model showed increased apoptosis and cardiac myocytes from *Ckmm‐Cre* ^*+/‐*^ *;Ercc1* ^*−/fl*^ mice were hypersensitive to genotoxins, resulting in apoptosis. p53 levels and target gene expression, including several antioxidants, were increased in cardiac tissue from *Ckmm‐Cre* ^*+/−*^ *;Ercc1* ^*−/fl*^ and *Ercc1* ^−/D^ mice. Despite this, cardiac tissue from older mutant mice showed evidence of increased oxidative stress. Genetic or pharmacologic inhibition of p53 attenuated apoptosis and improved disease markers. Similarly, overexpression of mitochondrial‐targeted catalase improved disease markers. Together, these data support the conclusion that DNA damage produced endogenously can drive cardiac disease and does so mechanistically via chronic activation of p53 and increased oxidative stress, driving cardiac myocyte apoptosis, dilated cardiomyopathy, and sudden death.

## Graphical abstract

With the loss of Ercc1, endogenous DNA damage is not repaired, leading to activation of p53. In response to chronic genotoxic stress, p53 activation overcomes antioxidant defenses, inducing cardiac myocyte apoptosis, leading to dilated cardiomyopathy and death. Either genetic or pharmacologic inhibition of p53 attenuates cardiac myocyte death and ventricular remodeling.

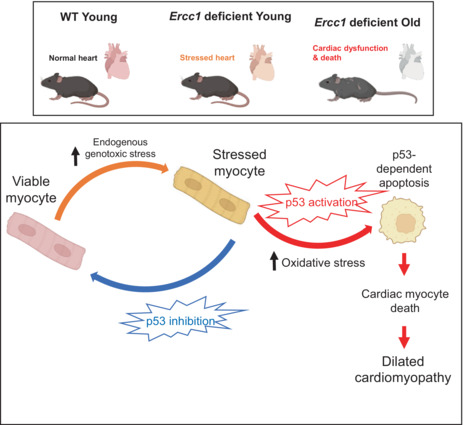

**Abbreviations**
AoV: aortic velocity
Ckmm‐Cre: muscle‐specific creatinine kinase Cre
cTnI: cardiac troponin
CO: cardiac output
DNA: deoxyribonucleic acid
E/A: the ratio of the E to A wave – a measure of peak velocity blood flow
E: E wave velocity – the peak velocity blood flow from the LV relaxation in early diastole
E/E’: the ratio between early mitral inflow and mitral annular early diastolic velocities
EDV: end diastolic volume
ESV: end systolic volume
EF: ejection fraction
FS: fractional shortening
GSH: Glutathione
GSSG: Glutathione disulfide
HR: heart rate
IVS: interventricular septum thickness
LVESD: left ventricular end systolic diameter
LVEDD: left ventricular end diastolic diameter
LVID: left ventricular internal diameter
LVPW: left ventricular posterior wall thickness
MCP‐1: monocyte chemoattractant protein‐1
NER: nucleotide excision repair
TNF‐α: tumor necrosis factor‐α
ROS: reactive oxygen species
mitCAT: Mitochondrial targeted human catalase
SV: stroke volume

## 1 INTRODUCTION

DNA damage and activation of the DNA damage response are implicated in promoting myocardial failure (Bartunek et al., 2002; Higo et al., 2017; Shukla et al., 2011). Furthermore, genotoxic stress caused by radiation (Darby et al., 2010; Plummer et al., 2011) or cancer chemotherapeutic drugs (Fulbright, 2011; Shakir & Rasul, 2009) promotes cardiomyopathy. However, the contribution of spontaneous, endogenous DNA damage, accrued through normal metabolic processes, to heart disease has not been elucidated.

To address this gap in knowledge, we used murine models where the DNA repair gene *Ercc1* was deleted. ERCC1‐XPF is a heterodimeric DNA repair endonuclease required for nucleotide excision repair (NER) of bulky, helix‐distorting DNA lesions, repair of DNA interstrand crosslinks, and the repair of some double‐strand breaks (Gregg et al., 2011). We demonstrated previously that systemic reduction in the level of ERCC1‐XPF in mice impairs DNA repair, increases the level of spontaneous oxidative DNA damage, senescent cell accumulation, and age‐related pathology in multiple organ systems (Dollé et al., 2011; Goss et al., 2011; Gregg et al., 2012; Lavasani et al., 2012; Robinson et al., 2018; Tilstra et al., 2014; Wang et al., 2012). Reduced expression of ERCC1‐XPF in humans can cause a progeroid syndrome affecting many organ systems (Dollé et al., 2006).

To test the hypothesis that endogenous DNA damage contributes to cardiac and skeletal muscle dysfunction and disease, we assessed the cardiac and skeletal muscle phenotypes in mice in which *Ercc1* expression was genetically depleted in differentiated striated myocytes using a muscle‐specific creatinine kinase Cre (*Ckmm*‐Cre) driver (Brüning et al., 1998) in conjunction with a floxed *Ercc1* allele (Yousefzadeh et al., 2021). In addition, cardiac tissue from *Ercc1* ^*−*/D^ hypomorphic mice, in which the gene is depleted systemically, was examined (Gregg et al., 2011; Robinson et al., 2018). *Ckmm‐Cre* ^*+/−*^ *;Ercc1* ^*−/fl*^ mice died spontaneously by 7 months of age with severe dilated cardiomyopathy. Hearts of young adult mutant mice (2–3‐month‐old) were structurally and functionally normal, but by 6‐months‐of‐age, the mutant mice displayed morphologic, functional, and molecular markers of dilated cardiomyopathy, indicative of a degenerative disease process. Similarly, dilated cardiomyopathy frequently occurs during accelerated aging, driven by, for example, viral infection, cancer chemotherapy, and diabetes (Bloom et al., 2016; Prandi et al., 2022; Tschöpe et al., 2021) At the cellular level, cardiac myocytes isolated from mice with myocyte‐specific or systemic depletion of *Ercc1* expression were hypersensitive to genotoxic stress and prone to apoptosis via a p53‐dependent mechanism. In vivo, disease phenotypes were partially rescued by depleting *p53* expression or overexpression of mitochondrial‐targeted catalase to reduce oxidative stress. Taken together, these data indicate that spontaneous, endogenous DNA damage induces cardiac myocyte loss, development of significant cardiac pathology, and premature death.

## 2 RESULTS

### 2.1 Deletion of *Ercc1* in striated muscle leads to premature death

**FIGURE 1:**
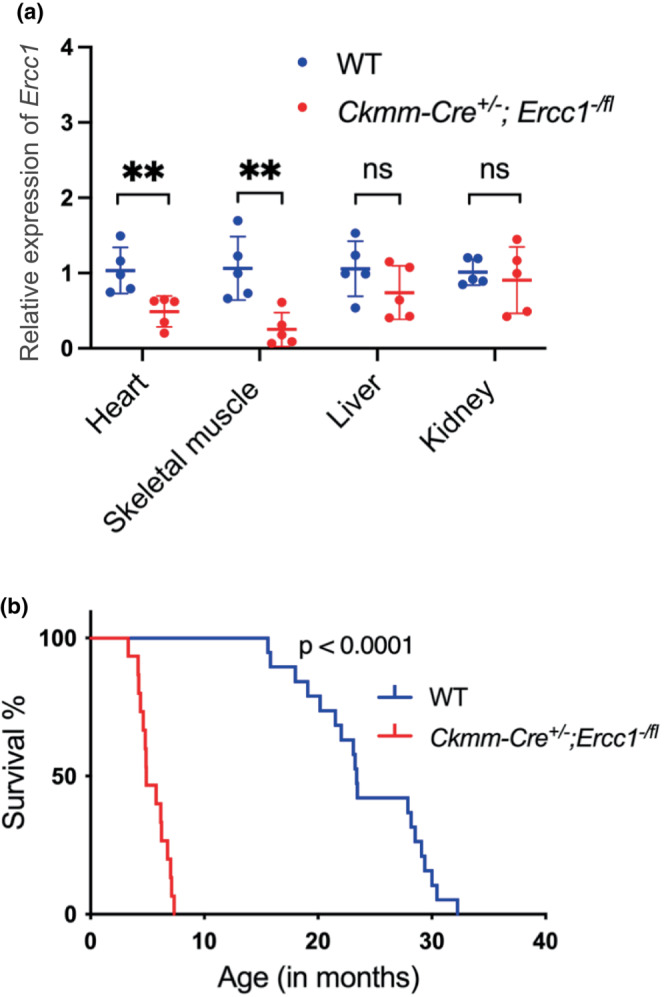
Deletion of *Ercc1*, a DNA repair gene, in striated muscle leads to premature death. (a) qRT‐PCR measurement of *Ercc1* mRNA levels in the heart, skeletal muscle, liver, and kidney from the tissue specific‐mutant *Ckmm‐Cre* ^*+/−*^ *;Ercc1* ^*−/fl*^ mice and wild‐type (WT) animals. See Table S4 for primer sets. See Table S5 for detailed relative expression levels of target genes. Data represent the mean ± SD (*n* = 5 mice, 2–3 months old), two‐tailed, paired Student' s *t*‐test, **p < 0.01, ns = non‐significant. The mRNA expression unit is fold change normalized to the control group. (b) Kaplan–Meier survival curve demonstrating that *Ckmm‐Cre* ^*+/−*^ *;Ercc1* ^*−/fl*^ mice have a significantly reduced lifespan (*n* = 15 mutant mice, median lifespan 4.9 months) compared to WT animals (*n* = 19, median lifespan 23.4 months), *p* < 0.0001, Log‐rank (Mantel‐Cox) test. See Figure S3 for sex‐specific differences in lifespan.

*Ckmm‐Cre* ^*+/−*^ *;Ercc1* ^*−/fl*^ mice were generated by crossing inbred C57BL/6J *Ckmm‐Cre* ^+/−^; *Ercc1* ^+/−^ mice with inbred FVB/n *Ercc1* ^*+/fl*^ mice (Figure S1). Littermate mice were used as controls for all studies, including animals heterozygous for the *Cre* transgene, heterozygous for the floxed or knock‐out *Ercc1* allele, and combinations thereof, as well as age‐matched wild‐type mice (WT), all in an F1 hybrid genetic background. *Cre* recombinase expression in differentiated striated myocytes begins at Day 13 of embryonic development (Wang et al., 1999). Induction of *Ercc1* deletion in adult animals would be preferable to study the health impact of a hallmark of aging (i.e., increased genotoxic stress). However, tamoxifen induces DNA damage (Carthew et al., 1995) and we observed that tamoxifen is lethal to *Ercc1*‐deficient mice and cells (data not shown), necessitating constitutive expression of *Cre* recombinase from the *Ckmm* promoter, which is activated at E17 and peaks at postnatal Day 10 (Agrawal et al., 2012). Mutant mice were born at Mendelian frequency (Table S1) with no apparent developmental abnormalities (i.e., no difference in morphology, size, or weight). *Ercc1* expression was significantly attenuated in cardiac and skeletal muscle at the transcriptional level, whereas no reduction in expression was observed in liver and kidney, tissues in which *Cre* recombinase was not expressed (Figure 1a). *Ercc1* expression was significantly lower in cardiac tissue of *Ercc1* ^*−/D*^ mice relative to *Ckmm‐Cre* ^*+/−*^ *;Ercc1* ^*−/fl*^ mice (Figure S2a), the former of which have systemic depletion of *Ercc1* expression (Dollé et al., 2011) and were used as a second genetic model of DNA repair deficiency to confirm the results in the tissue‐specific *Ercc1* deletion model (see the accompanying manuscript by de Boer et al. (2023) describing the cardiac phenotype in *Ercc1* ^*−/D*^ mice). ERCC1 protein was also reduced in cardiac tissue of mutant animals (Figure S2b). The loss of ERCC1‐XPF expression in cardiac tissue led to premature death in mice (Figure 1b) with a median lifespan of 4.9 months (vs. 23.4 months for wild‐type (WT) mice) and a maximum lifespan of 7 months (vs. 32.3 months in WT mice).

Notably, maximum lifespan was significantly greater in female mice compared to male (Figure S3). Body weight, body composition, and lean mass were not significantly different between mutant animals and age‐matched WT controls, although there was a significant increase in fluid in 2–3‐month‐old mutant animals compared to controls (Figure S4). To determine whether skeletal muscle integrity was impacted by deletion of *Ercc1*, we examined the gastrocnemius. Although *Ercc1* was successfully knocked‐out in skeletal muscle (Figure 1a), no histological differences were detected in the skeletal muscle of 5–6‐month‐old *Ckmm‐Cre* ^*+/‐*^ *;Ercc1* ^*−/fl*^ compared to WT littermates (Figure S5a). Furthermore, there was no impact on PAX7^+^ satellite cell density (Figure S5b,c) or muscle stem/progenitor cell proliferation/differentiation ex vivo (Figure S5d,e). Accordingly, muscle regeneration in mutant animals after deliberate injury was not impaired (Figure S5f–h). Finally, there was only a modest reduction in grip strength in older mutant mice compared to controls (Figure S5i). These data indicate that it is unlikely that skeletal muscle pathology contributed to the premature death of *Ckmm‐Cre* ^*+/−*^ *;Ercc1* ^*−/fl*^ mice.

### 2.2 Deletion of *Ercc1* in striated muscle leads to spontaneous dilated cardiomyopathy

**FIGURE 2:**
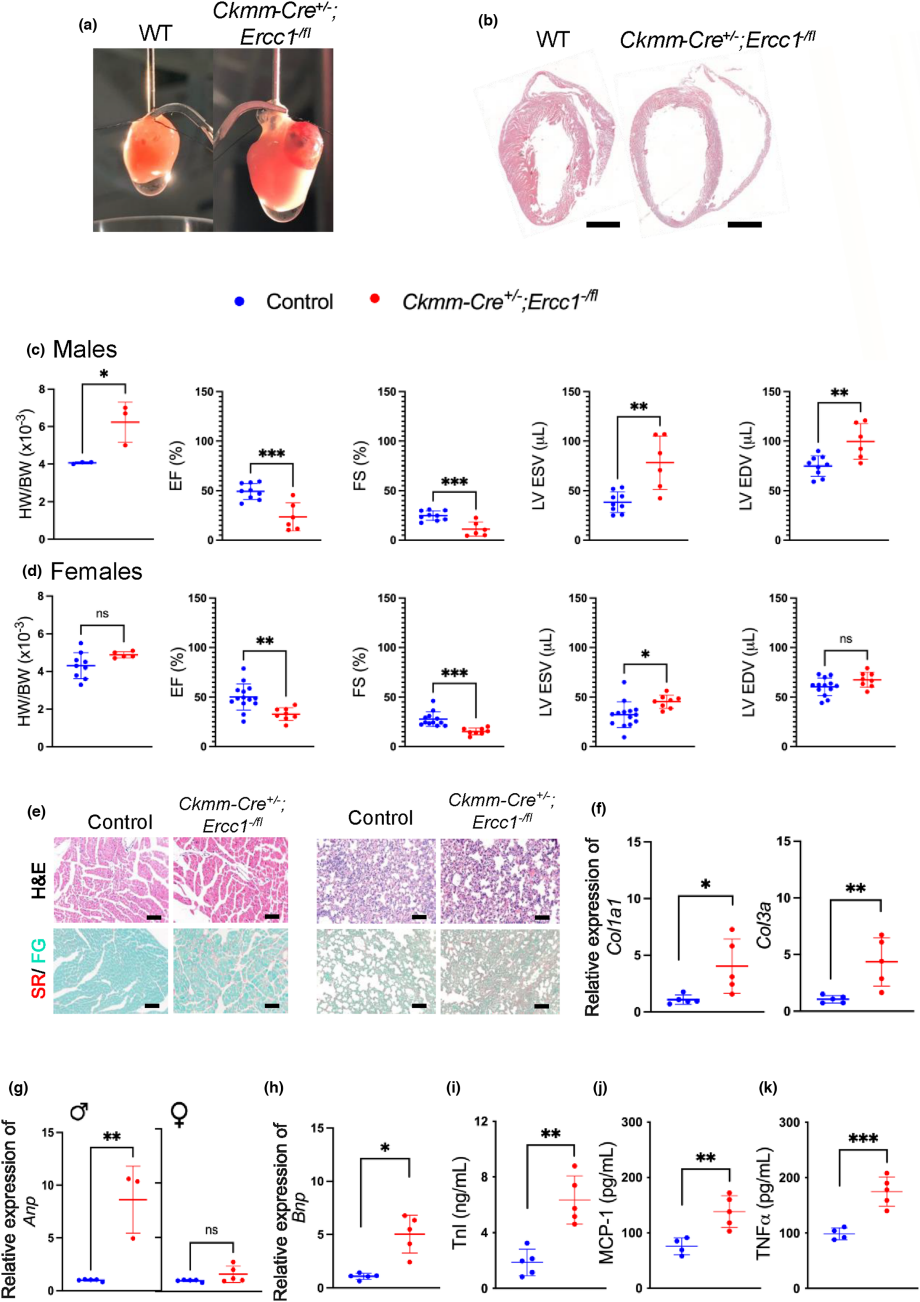
Deletion of *Ercc1* in striated muscle leads to dilated cardiomyopathy. (a) Representative images of hearts from *Ckmm‐Cre* ^*+/−*^ *;Ercc1* ^*−/fl*^ and WT mice at 6‐months of age (Scale bar: 2 mm). (b) Representative images of H&E stained sagittal section of the heart from a *Ckmm‐Cre* ^*+/−*^ *;Ercc1* ^*−/fl*^ and WT at 6 months of age. (Scale bar: 2.5 mm). (c) Measures of heart weight to body weight ratio and cardiac function (ejection fraction (EF), fractional shortening (FS), left ventricular end‐systolic volume (LV ESV), and left ventricular end‐diastolic volume (LV EDV)) calculated from echocardiography data from male mutant mice and littermate controls at 6 months of age. Graphs indicate the mean with error bars representing ± SD (*n* = 6–9 mice per group); two‐tailed, unpaired Student's *t*‐test *p < 0.05, **p < 0.01, ***p < 0.001. (d). Same as c for female mice at 6 months of age (controls *n* = 14 and *Ckmm‐Cre* ^*+/−*^ *;Ercc1* ^*−/fl*^ *n* = 8 mice). A summary of all echo data can be found in Table S2. (e) Representative images of heart (left) and lung (right) sections stained with H&E or picrosirius red with Fast Green counterstain (SR/FG) to detect fibrosis. *Ckmm‐Cre* ^*+/−*^ *;Ercc1* ^*−/fl*^ mice and littermate controls were 6 months of age. (Scale bar: 50 μM). (f) Measurement of collagen 1a1 (*Col1a1*), Collagen 3a (*Col3a*), (g) atrial natriuretic peptide (*Anp*), and (h) brain natriuretic peptide (*Bnp*) in left ventricular tissue of *Ckmm‐Cre* ^*+/−*^ *;Ercc1* ^*−/fl*^ mice and WT controls by qRT‐PCR. Data represent the mean ± SD (*n* = 3–5 mice per group, 6 months of age), Two‐tailed, unpaired Student's *t*‐test *p < 0.05 and **p < 0.01. See Table S5 for detailed relative expression of target genes. The mRNA expression unit is fold change normalized to the control group. (i) Serum cardiac troponin (Tnl) measured by ELISA. Data represent the mean ± SD, (*n* = 5 mice per group; 6 months of age), two‐tailed, unpaired Student's *t*‐test **p < 0.01. (j) Monocyte chemoattractant protein (MCP‐1) and (k). Tumor necrosis factor‐a (TNF‐a) in serum from *Ckmm‐Cre* ^*+/−*^ *;Ercc1* ^*−/fl*^ mice, measured by ELISA. Data represent the mean ± SD. (*n* = 4–5 per group; 4–6 months of age), two‐tailed, unpaired Student's *t*‐test **p < 0.01 and ***p < 0.001.

In contrast to the skeletal muscle, deletion of *Ercc1* in cardiac myocytes had a profound effect on cardiac muscle. The hearts of 6‐month‐old *Ckmm‐Cre^+/‐^;Ercc1* ^*−/fl*^ mice were enlarged compared to littermate controls (Figure 2a). Histological examination revealed left ventricular wall thinning and ventricular dilation (Figure 2b), which was accompanied by a significant increase in cardiac myocyte cross‐sectional area (Figure S6). The cardiac myocyte hypertrophy suggests cardiac myocyte loss in the mutant mice. In male but not female *Ckmm‐Cre^+/‐^;Ercc1* ^*−/fl*^ mice, the heart weight/body weight ratio was significantly increased (Figure 2c,d first panels and Figure S7) consistent with myocardial hypertrophy and male mice having a more severe phenotype than female (Figure S3). The size of young (2–3‐month‐old) mutant mouse heart was indistinguishable from controls (Figure S8), suggesting that the ventricular enlargement in the older mutant animals was due to a degenerative disease process rather than a developmental defect.

In addition to the significant cardiac structural defects noted in the *Ckmm‐Cre^+/‐^;Ercc1* ^*−/fl*^ mice at 6 months, cardiac function in these mice was significantly impaired. Of note, we used several control mice to account for potential effects of the different genetic manipulations in our model, including *Ercc1* ^*−/fl*^, *Ckmm‐Cre* ^*+/−*^, and *Ercc1* ^*+/−*^ mice. There were no significant differences in systolic function (ejection fraction, EF, and fractional shortening, FS) between these control groups, all of which have at least one functional allele of *Ercc1* (Figure S9). However, in the *Ckmm‐Cre* ^*+/−*^ *;Ercc1* ^*−/fl*^ mice, at 6 months of age, there was a significant impairment in systolic dysfunction (EF, FS) in both male and female mice compared to the age‐matched control mice (Figure 2c males and Figure 2d females; Table S2). Consistent with systolic dysfunction, left ventricular end‐systolic volume (LVESV) was also significantly increased in both male and female *Ckmm‐Cre* ^*+/−*^ *;Ercc1* ^*−/fl*^ mice relative to control mice. Left ventricular end‐diastolic volume (LVEDV) was significantly increased in male but not female *Ckmm‐Cre* ^*+/−*^ *;Ercc1* ^*−/fl*^ mice compared to control animals, indicating ventricular dilation in the males. The sex‐specific differences in LVEDV and lifespan (Figure S3) suggest acceleration of heart failure in male mice relative to female, which is commonly seen in murine models of heart disease and is attributed at least in part to the protective effect of estrogen (Du, 2004). Importantly, cardiac function was indistinguishable between *Ckmm‐Cre* ^*+/−*^ *;Ercc1* ^*−/fl*^ and control mice at 3–4 months of age, further supporting a degenerative disease process (Figure S10).

Additionally, deletion of *Ercc1* in cardiac myocytes induced marked ventricular interstitial fibrosis at 6 months in *Ckmm‐Cre* ^*+/−*^ *;Ercc1* ^*−/fl*^ (Figure 2e), consistent with histopathologic changes in dilated cardiomyopathy (Khan & Sheppard, 2006; Li et al., 2018; Schulze, 2009). *Collagen 1a1* and *Collagen 3a* expression were also significantly increased in cardiac tissue from *Ckmm‐Cre* ^*+/−*^ *;Ercc1* ^*−/fl*^ mice at 6 months, but not at 2–3 months (Figure 2f and Figure S11a,b). Interestingly, *Ckmm‐Cre* ^*+/−*^ *;Ercc1* ^*−/fl*^ mice showed significant fibrotic changes in pulmonary vasculature at 6 months (Figure 2e, right panels), commonly seen in cardiomyopathy (Lijima et al., 1993) and consistent with our observation that mutant animals displayed dyspnea prior to sudden death (Videos S1 and S2).

Expression of heart failure markers *Anp* and *Bnp* was increased in heart tissue of male *Ckmm‐Cre* ^*+/−*^ *;Ercc1* ^*−/fl*^ mice at 6‐months‐of‐age, but not in 2–3‐month‐old animals (Figure 2g,h and Figure S11c,d). Additionally, cardiac troponin (cTnI), a cardiac‐specific enzyme used as a marker of cardiac damage, (Adams et al., 1993; Missov et al., 1997; O'Brien et al., 2006) was significantly increased in serum of *Ckmm‐Cre* ^*+/−*^ *;Ercc1* ^*−/fl*^ mice at 6 months of age (Figure 2i) but not 2–3 months of age (Figure S11e). Interestingly, inflammatory markers monocyte chemoattractant protein‐1 (MCP‐1) and tumor necrosis factor‐α (TNF‐α), associated with age‐related diseases including cardiac disease, (Bellisarii et al., 2001; Niu & Kolattukudy, 2009) were also elevated in the serum of *Ckmm‐Cre* ^*+/−*^ *;Ercc1* ^*/fl*^ mice at 6 months compared to age‐matched controls and no change in 2–3 months of age mice (Figure 2j,k and Figure S11f,g). In summary, the changes in ventricular structure and function of *Ckmm‐Cre* ^*+/−*^ *;Ercc1* ^*−/fl*^ mice indicate that loss of the ability to repair endogenous DNA damage in cardiac myocytes leads to a severe, spontaneous, and early onset of dilated cardiomyopathy.

### 2.3 Deletion of *Ercc1* in cardiac myocytes causes increased p53, sensitivity to genotoxins, and apoptosis

**FIGURE 3:**
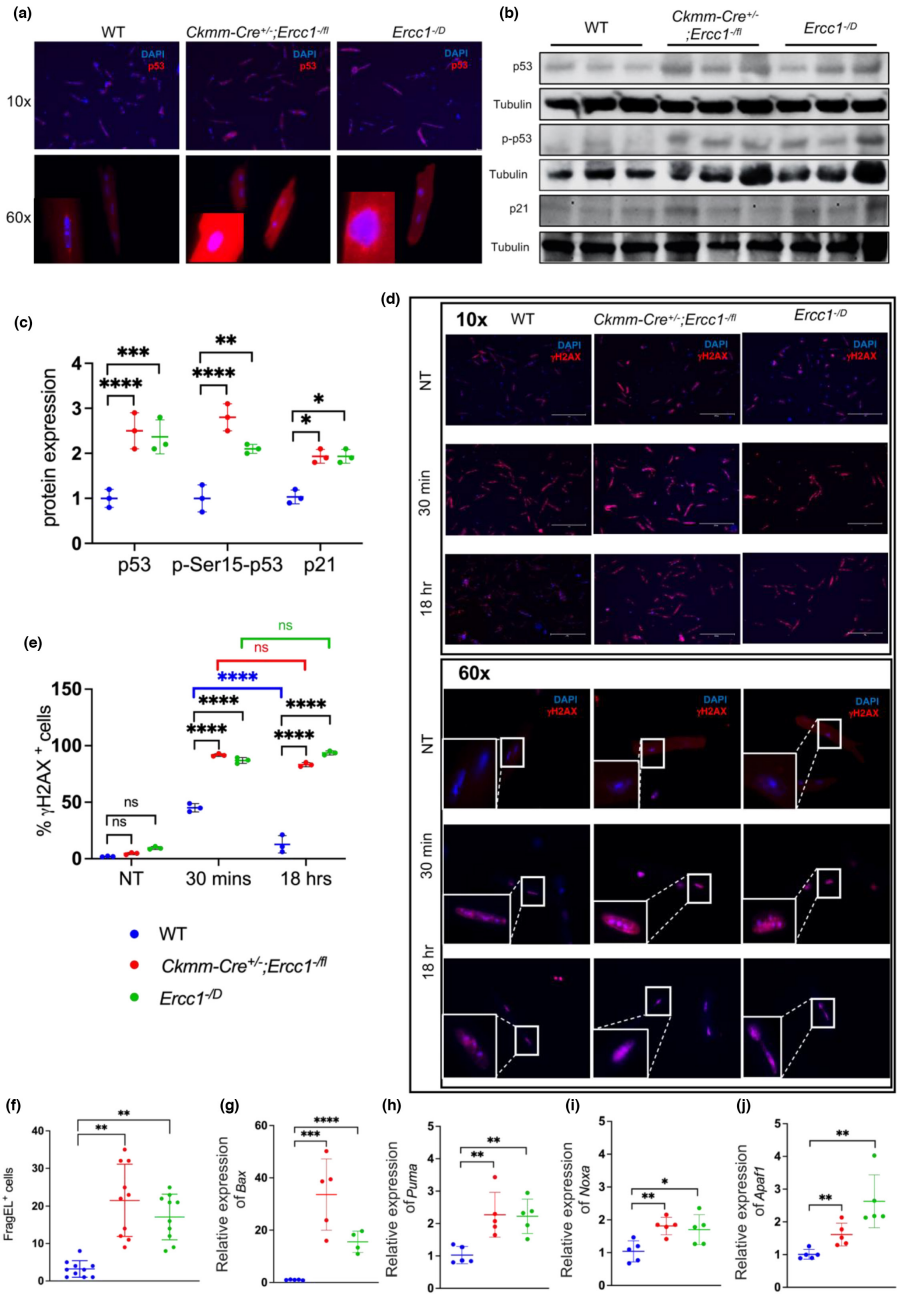
Deletion of *Ercc1* in striated muscle induces elevated levels of p53, markers of DNA damage, and apoptosis in isolated cardiac myocytes. (a) Immunocytochemical detection of p53 in cardiac myocytes isolated from 3‐month‐old WT, *Ckmm‐Cre* ^*+/−*^ *;Ercc1* ^*−/fl*^ and *Ercc1* ^*/D*^ mice, which have tissue‐specific or systemic depletion of *Ercc1* expression, respectively. (b) Protein lysates from 6‐month‐old mouse hearts of indicated genotypes were analyzed by immunoblot to detect p21, p53, or (phospho) p‐Ser15‐p53. Tubulin was used as a loading control. See Figure S16 for uncropped immunoblots. (c) Quantification of protein levels in b. One‐way ANOVA with Tukey's multiple comparison test, *p < 0.05; **p < 0.01, ***p < 0.001, ****p < 0.0001. (d) Representative images of immunofluorescence detection of γH2AX in doxorubicin‐treated (2.5 μM) cardiac myocytes isolated from 3‐month‐old WT, *Ckmm‐Cre* ^*+/‐*^ *;Ercc1* ^*−/fl*^ and *Ercc1* ^*−/D*^ mice at two time points post‐drug treatment. (Scale bar: 40 μM; NT = not treated). (e) Graphic representation of the fraction of γH2AX foci‐positive cells/field. Five independent fields per biological replicate were used for counting. Data represent the mean ± SD. (*n* = 3 biological replicates per genotype), two‐way ANOVA (mixed model, repeated measures) with Tukey's multiple comparison test was used. ****p < 0.0001; ns = non‐significant. (f) The number of FragEL‐positive apoptotic cells per field was measured on 10 consecutive tissue sections from hearts of mice of the indicated genotypes. Graphed is the mean ± SD, *n* = 5 animals per genotype, one‐way ANOVA **p < 0.001. (g–k) Relative expression of *Bax Puma*, *Noxa*, and *Apaf1*, pro‐apoptotic p53 target genes, in cardiac tissue from mice of the indicated genotypes measured by qRT‐PCR. See Table S4 for primer sets. See Table S5 for detailed relative expression of target genes. Data represent the mean ± SD. *n* = 5 animals per genotype One‐way ANOVA with Tukey's multiple comparison test, *p < 0.05; **p < 0.01, ***p < 0.001, ****p < 0.0001. The mRNA expression unit is fold changes normalized to the control group.

Exogenous or environmental DNA damage can induce cardiac myocyte apoptosis (Higo et al., 2017; Shukla et al., 2011). Given that p53 is a key regulator of cell death in response to genotoxic stress (Roos & Kaina, 2006), we performed immunocytochemical detection of p53 in cardiac myocytes isolated from 3‐ to 4‐month‐old mice (Figure 3a). Indeed, p53 was elevated in mutant cardiac myocytes compared to WT myocytes. We also measured p53, activated p53 (phosphoSer15‐p53), and a downstream effector of p53 activation, p21, by immunoblotting in cardiac tissue from 4‐ to 6‐month‐old *Ckmm‐Cre* ^*+/−*^ *;Ercc1* ^*−/fl*^ and *Ercc1* ^*−/D*^ mice as well as age‐matched control mice (Figure 3b). Levels of p53, pSer15‐ p53, and p21 were elevated in *Ckmm‐Cre* ^*+/−*^ *;Ercc1* ^*−/fl*^ and *Ercc1* ^*−/D*^ versus control hearts at 4–6 months of age (Figure 3c). Finally, immunofluorescence detection of p53 in phalloidin‐stained cardiac muscle sections revealed increased p53^+^ nuclei in cardiac myocytes from *Ckmm‐Cre* ^*+/−*^ *;Ercc1* ^*−/fl*^ mice in vivo (Figure S12).

To establish whether loss of *Ercc1* conferred increased sensitivity to genotoxic stress, we cultured adult cardiac myocytes from *Ckmm‐Cre* ^*+/−*^ *;Ercc1* ^*−/fl*^ and *Ercc1* ^*−/D*^ mice (~15‐weeks‐old) and exposed them to genotoxic stress from doxorubicin, a genotoxin known to mediate cardiac damage (Kim et al., 2018; Mitry & Edwards, 2016; Swain et al., 2003). Phosphorylated histone 2AX (γH2AX foci) was measured by immunofluorescence as a marker of DNA damage (Figure 3d) (Cordelli & Paris, 2013; Firsanov et al., 2011; Mah et al., 2010). Doxorubicin robustly and rapidly increased γH2AX foci indicating significant DNA damage in cardiac myocytes from *Ckmm‐Cre* ^*+/−*^ *;Ercc1* ^*−/fl*^ and *Ercc1* ^*−/D*^ mice, as well as WT cardiac myocytes. While DNA damage had resolved in WT myocytes at 18 h, as indicated by a significant reduction in γH2AX foci, the foci persisted in *Ckmm‐Cre* ^*+/−*^ *;Ercc1* ^*−/fl*^ and *Ercc1* ^*−/D*^ myocytes, confirming loss of DNA repair upon deletion of *Ercc1* (Figure 3d,e). We next investigated whether p53 activation in the DNA repair‐deficient mice led to increased expression of markers associated with cellular senescence or apoptosis. No increase in expression of *p16* ^*Ink4a*^, *p21* ^*Cip1*^, or numerous senescence‐associated secretory phenotype genes was detected in cardiac tissue of *Ckmm‐Cre* ^*+/−*^ *;Ercc1* ^*−/fl*^ mice relative to wild‐type animals as measured by qRT‐PCR (data not shown). Similarly, no increase in senescence‐associated βgalactosidase activity was detected in heart tissue or isolated cardiomyocytes from *Ckmm‐Cre* ^*+/‐*^ *;Ercc1* ^*−/fl*^ mice. In contrast, we did find an increase in apoptotic cells in both *Ckmm‐Cre* ^*+/−*^ *;Ercc1* ^*/fl*^ and *Ercc1* ^*−/D*^ hearts relative to control hearts at 6 months of age by FragEL fluorescence staining (Figure 3f). Further, the mRNA of four pro‐apoptotic transcriptional targets of p53, *Bax*, *Puma*, *Noxa*, *and Apaf1* (Nakano & Vousden, 2001; Pawlowski & Kraft, 2000) was significantly increased in *Ckmm‐Cre* ^*+/−*^ *;Ercc1* ^*−/fl*^ and *Ercc1* ^*−/D*^ murine heart tissue relative to age‐matched control animals (Figure 3g–j). Notably, expression of *Bax* and *Puma* were not elevated in tissues from 2‐ to 3‐month‐old *Ckmm‐Cre* ^*+/−*^ *;Ercc1* ^*−/fl*^ and *Ercc1* ^*−/D*^ mice, relative to age‐matched controls (Figure S13), associating apoptosis with the onset of frank disease.

### 2.4 Apoptosis of DNA repair‐deficient cardiac myocytes is p53 dependent

**FIGURE 4:**
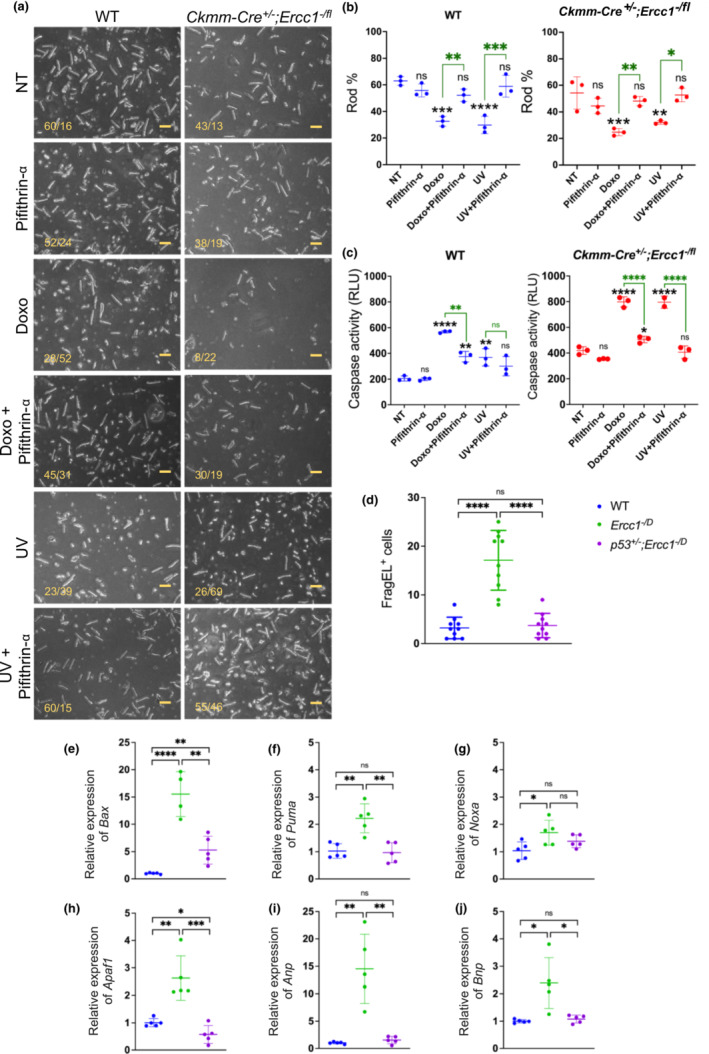
Apoptosis of *Ercc1*‐deficient cardiac myocytes is p53‐dependent. (a) Representative images of cardiac myocytes isolated from 3‐month‐old mice of the indicated genotypes, which were not treated (NT), or with treated with Pifithrin‐α (an inhibitor of p53) treated, doxorubicin (a genotoxin), doxorubicin + Pifithrin‐α, UV, or UV+ Pifithrin‐α for 24 h and assessed for cell viability (rods = viable vs. rounds = dying or dead; numbers reported for each image in bottom left) (Scale bar: 100 μM). (b) Quantification of rod‐shaped cardiac myocytes (%) for the indicated genotypes and treatments groups. Data represent the mean ± SD, *n* = 3 animals per genotype, two‐way ANOVA in mutant mice and WT mice. *p < 0.05; **p < 0.01; ***p < 0.001; ****p < 0.0001 and ns = non‐significant. Black font for ns (non‐significant) and * denotes the comparison of a treated group to the non‐treated group (NT). Green font and bars denote the comparison between cells treated with a genotoxin +/− the p53 inhibitor. (c) Caspase activity in cardiac myocytes for the indicated genotypes and treatment groups. Apoptosis was measured 24 h postdoxorubicin treatment using a Caspase 3/7 Glow assay. Graphed is the mean ± SD, *n* = 3 animals per genotype, two‐way ANOVA in mutant mice and WT mice. *p < 0.05; **p < 0.01; ***p < 0.001; ****p < 0.0001 and ns = non‐significant. Black font for ns and * denotes the comparison of treated group to the non‐treated group (NT) and green font represents the comparison between cardiac myocytes treated with a genotoxin ± the p53 inhibitor. (d) The number of FragEL‐positive cardiac myocytes per field was measured on heart sections from mouse tissues of the indicated genotypes. Graphed is the mean ± SD, *n* = 9 animals per genotype, one‐way ANOVA ****p < 0.0001. (e–h) Relative expression of *Bax*, *Puma*, *Noxa*, and *Apaf1*, pro‐apoptotic p53 target genes, in cardiac tissues of mice of the indicated genotypes measured by qRT‐PCR. Graphed is the mean ± SD, *n* = 5 animals per genotype, one‐way ANOVA with Tukey's multiple comparison test, *p < 0.05, **p < 0.01, ***p < 0.001, ****p < 0.0001, and ns = non‐significant. The mRNA expression unit is fold changes normalized to the control group. (i, j) Relative expression of *Anp* and *Bnp*, heart failure markers, in cardiac tissue of mice of the indicated genotypes measured by qRT‐PCR. See Table S4 for primers and Table S5 for detailed relative expression of target genes. Data represent the mean ± SD, *n* = 5 animals per genotype, one‐way ANOVA with Tukey's multiple comparison test, *p < 0.05, **p < 0.01, and ns = non‐significant. The mRNA expression unit is fold changes normalized to the control group.

To establish whether p53 activation drives death of cardiac myocytes in response to genotoxic stress, cultured adult cardiac myocytes from *Ckmm‐Cre* ^*+/−*^ *;Ercc1* ^*−/fl*^ and WT mice were treated with doxorubicin or UV radiation to induce DNA damage in vitro in the presence or absence of the p53 inhibitor, Pifithrin‐α, and cell death was quantified. Cardiac myocyte death was quantified by cell morphology (rod = healthy cardiac myocyte; round = dying/dead; Figure S14). Of note, UV‐induced DNA damage is only repaired by nucleotide excision repair in mammalian cells, which is an ERCC1‐dependent mechanism (Shah & He, 2015; Sinha & Häder, 2002). Thus, *Ckmm‐Cre* ^*+/−*^ *;Ercc1* ^*−/fl*^ cardiac myocytes are expected to be hypersensitive to UV. UV irradiation increased cardiac myocyte death, as measured by cell morphology in both *Ckmm‐Cre* ^*+/−*^ *;Ercc1* ^*−/fl*^ mice and WT cardiac myocytes (Figure 4a,b; Table S3). Importantly, both doxorubicin and UV increased cell death in *Ckmm‐Cre* ^*+/−*^ *;Ercc1* ^*−/fl*^ cardiac myocytes to a greater extent than in WT cells (*p* = 0.04 for doxorubicin and *p* = 0.04 for UV when comparing the percent rods in WT vs. *Ckmm‐Cre* ^*+/−*^ *;Ercc1* ^*−/fl*^ cardiac myocytes exposed to genotoxic stress). Cell death was rescued by pharmacological inhibition of p53 with pifithrin‐α. Notably, caspase activity, a marker of apoptosis, was significantly greater in untreated *Ckmm‐Cre* ^*+/−*^ *;Ercc1* ^*−/fl*^ cardiac myocytes compared to WT (*p* = 0.0005) (Figure 4c). Doxorubicin and UV induced a significant increase in cardiac myocyte caspase activity in both genotypes, but this was significantly greater in *Ckmm‐Cre* ^*+/−*^ *;Ercc1* ^*−/fl*^ cardiac myocytes compared to WT (*p* = 0.0006 for doxorubicin and *p* = 0.0047 for UV when comparing the percent rods in WT vs. *Ckmm‐Cre* ^*+/−*^ *;Ercc1* ^*−/fl*^ cardiac myocytes exposed to genotoxic stress). Again, pharmacologic inhibition of p53 activity reduced caspase activity to near baseline, in the *Ckmm‐Cre* ^*+/−*^ *;Ercc1* ^*−/fl*^ cardiac myocytes. These data confirm that the *Ercc1*‐deficient cardiac myocytes were DNA repair defective and establish that unrepaired DNA damage kills cardiac myocytes through a p53 dependent mechanism.

To further confirm these findings in vivo, we performed FragEL assay and found a significant increase in apoptotic cells in hearts from *Ercc1* ^*−/D*^ mice relative to control animals (Figure 4d), similar to hearts from *Ckmm‐Cre* ^*+/−*^ *;Ercc1* ^*−/fl*^ mice (Figure 3f). This was restored to baseline by genetic depletion of *p53* in *p53* ^*+/‐*^ *;Ercc1* ^*−/D*^ mice (Figure 4d). Additionally, expression of pro‐apoptotic transcriptional targets of p53, *Bax*, *Puma*, *Noxa*, *and Apaf1*, were significantly increased in cardiac tissues from *Ercc1* ^*−/D*^ mice relative to control animals (Figure 4e–h), similar to hearts from *Ckmm‐Cre* ^*+/−*^ *;Ercc1* ^*−/fl*^ mice (Figure 3g–j). This was again restored to baseline upon genetic depletion of p53 expression in *p53* ^*+/*^ *;Ercc1* ^*−/D*^ mice. Finally, *Anp* and *Bnp* expression (Figure 4i,j) were increased in heart tissue from *Ercc1* ^*−/D*^ mice, indicating tissue damage. This was largely mitigated in *p53* ^*+/‐*^ *;Ercc1* ^*−/D*^ mice. Thus, haploinsufficiency of p53 attenuated p53 activation, apoptosis, and expression of markers of heart failure, defining a mechanistic requirement for p53 in the pathologic remodeling of cardiac tissue under genotoxic stress.

We attempted to quantify endogenous oxidative DNA lesions (Wang et al., 2012) specifically repaired by nucleotide excision repair (Brooks et al., 2000) in cardiac tissue from *Ckmm‐Cre* ^*+/−*^ *;Ercc1* ^*−/fl*^ mice *and Ercc1* ^*−/D*^ mice. We used a highly sensitive and specific LCMS/MS/MS method including an isotopically labeled internal control. Surprisingly, the levels of four oxidative DNA lesions (cyclopurine adducts) were no different or significantly reduced in cardiac tissues from mutant animals compared to controls (Figure S15). We speculate that cells with high levels of DNA lesions in mutant cardiac tissues had undergone apoptosis, thus escaping detection. Thus, we interpret this as further evidence that cardiac myocytes are exquisitely vulnerable to apoptosis in response to genotoxic stress, including DNA damage produced endogenously.

### 2.5 Increased ROS in cardiac tissue of DNA repair‐deficient mice is p53‐dependent and pathogenic

**FIGURE 5:**
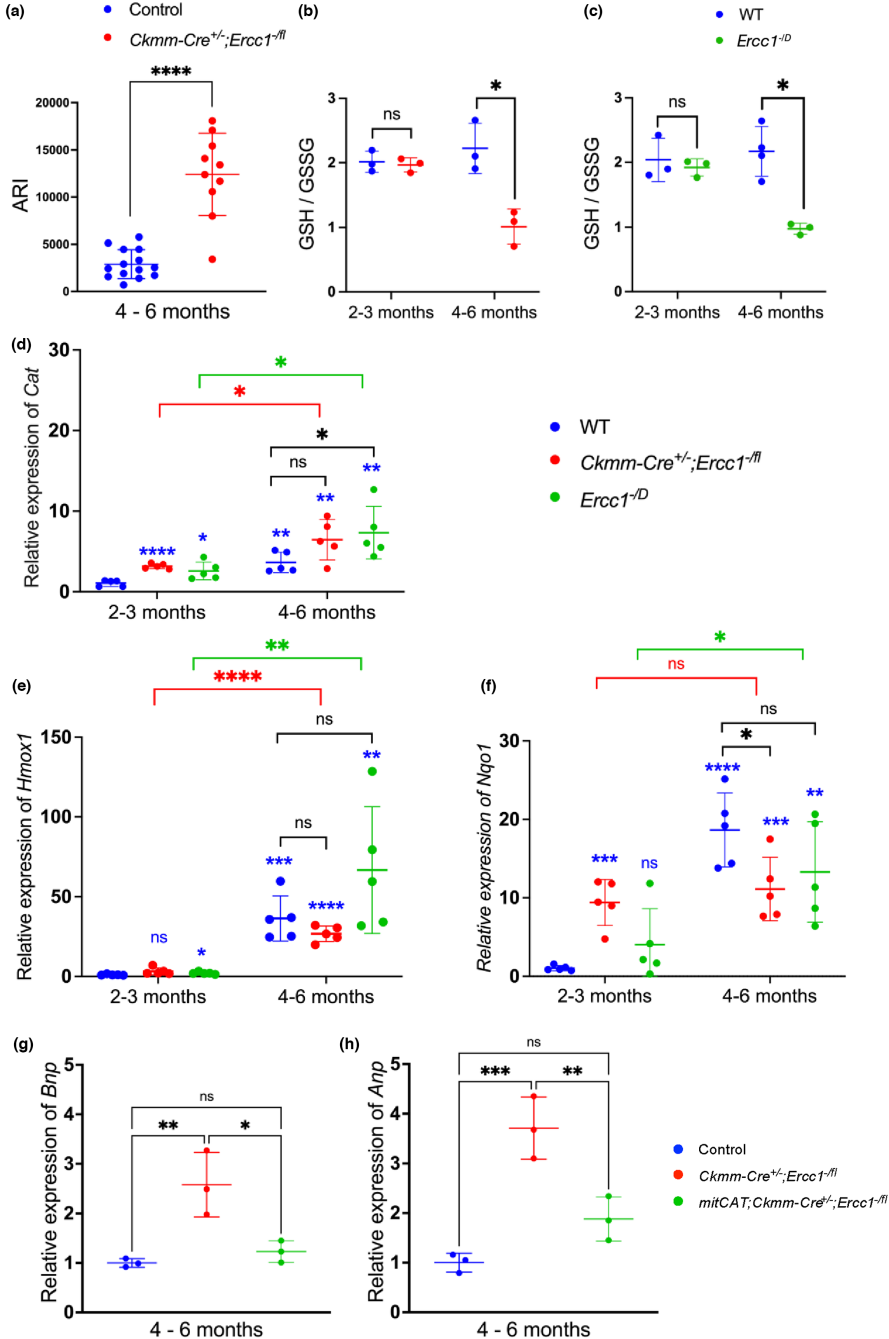
p53‐mediated oxidative stress contributes to heart failure in *Ercc1*‐deficient mice. (a) EPR detection of ascorbate free radical abundance (Asc^·−^) in 4‐ to 6‐month‐old *Ckmm‐Cre* ^*+/‐*^ *;Ercc1* ^*−/fl*^ mice compared to littermate controls. Data represent the mean ± SD, *n* = 10–14 per group, two‐tailed, unpaired Student's *t*‐test ****p < 0.0001. ARI, Ascorbyl Radical Intensity. (b, c) The ratio of reduced to oxidized glutathione (GSH/GSSG) in cardiac tissue from 2–3‐month‐old and 4‐ to 6‐month‐old *Ckmm‐Cre* ^*+/−*^ *;Ercc1* ^*−/fl*^ mice compared to littermate controls and in *Ercc1* ^−*/D*^ and WT mice of the same age. Values represent the mean ± SD, *n* = 3–4 per group, *p < 0.05 and ns = non‐significant determined by two‐tailed, unpaired Student's *t*‐test. (d–f) Relative expression of *Cat*, *Hmox1*, and *Nqo1*, antioxidant p53‐target genes, in cardiac tissue of mice of the indicated genotypes and age groups per above, measured by qRT‐PCR. The data represent the mean ± SD, *n* = 5 per group, two‐way ANOVA, *p < 0.05; **p < 0.001; ***p < 0.001; ****p < 0.0001 and ns = non‐significant. Blue asterisks indicate differences compared to young WT. The mRNA expression unit is fold changes normalized to the control group. (g, h) Relative expression of *Anp* and *Bnp* in LV of 4‐ to 6‐month‐old mice of the indicated genotypes measured by qRT‐PCR. Data represent the mean ± SD, *n* = 3 per group, one‐way ANOVA with Tukey's multiple comparison test, *p < 0.05, **p < 0.005, ***p < 0.001 and ns = non‐significant. See Table S5 for expression of target genes. The mRNA expression unit is fold changes normalized to the control group.

To define further the mechanism behind the genotoxic‐stress induced, p53‐dependent apoptosis of cardiac myocytes, we measured other pathways regulated by p53. There are several lines of evidence indicating p53 regulates ROS levels through regulation of NRF2, a transcription factor driving antioxidant gene expression in response to oxidative stress (Chen et al., 2013; Faraonio et al., 2006). NRF2 binds to antioxidant response/electrophile response elements to regulate the transcription of the antioxidant genes *Cat*, *Hmox1*, *and Nqo1* (Ade et al., 2009). Furthermore, we previously reported increased oxidative stress in tissues of *Ercc1* ^*−/D*^ mice due at least in part to reduced antioxidant buffering capacity (Robinson et al., 2018). Here, we observed a dramatic increase in ascorbate radical (Asc^·*−*^) abundance (a real‐time marker of ROS abundance in biologic samples; Jurkiewicz & Buettner, 1994; Sharma et al., 1994) in cardiac tissue of 4‐ to 6‐month‐old *Ckmm‐Cre* ^*+/−*^ *;Ercc1* ^*−/fl*^ mice compared to age‐matched controls (Figure 5a). Consistent with that, the ratio of GSH/GSSG was significantly decreased in cardiac tissue from the *Ckmm‐Cre* ^*+/−*^ *;Ercc1* ^*−/fl*^ and *Ercc1* ^*−/D*^ mutant mice at 4–6 months of age, indicative of increased oxidative stress and reduced antioxidant buffering capacity in the DNA repair‐deficient mice. No differences were observed between mutant and control mice at 2–3 months of age (Figure 5b,c). Additionally, mRNA levels of the three NRF2 target genes, *Cat*, *Hmox1*, and *Nqo1*, were more dramatically increased in old *Ercc1* ^*−/D*^ and *Cat* and *Hmox1*, in *Ckmm‐Cre* ^*+/−*^ *;Ercc1* ^*−/fl*^ mice compared to young (Figure 5d–f). These data illustrate that *Ercc1* deletion leads to increased oxidative stress in cardiac tissues of older mice.

Finally, to determine whether increased ROS contributes to cardiac failure in *Ckmm‐Cre* ^*+/−*^ *;Ercc1* ^*−/fl*^ mice, we generated DNA repair‐deficient mice expressing mitochondrial‐targeted human catalase (mitCAT) (Figure S1c). Catalase activity is increased 50‐fold in cardiac tissue of transgenic mice compared to WT mice (Schriner et al., 2005). Furthermore, mitCAT has been shown to improve cardiac function in aged WT mice (Schriner et al., 2005).

Overexpression of mitCAT in *Ckmm‐Cre* ^*+/−*^ *;Ercc1* ^*−/fl*^ mice attenuated expression of heart failure markers *Anp* and *Bnp* consistent with increased ROS contributing to disease pathogenesis (Figure 5g,h).

## 3 DISCUSSION

Multiple lines of evidence suggest that DNA repair is integral to cardiovascular health. Increased expression of the DNA repair proteins PCNA, DNA‐PKcs, and APE1 is correlated with left ventricular dysfunction in patients with idiopathic dilated cardiomyopathy, suggesting increased DNA repair activity in diseased cardiac tissue (Bartunek et al., 2002). In contrast, reduced expression of numerous DNA repair genes, including *ERCC1*, *XPA*, and *ATM*, are associated with stable angina and myocardial infarction (Zhang et al., 2017). There is at least one single nucleotide polymorphism in *ERCC1* significantly associated with coronary artery disease (Zhang et al., 2017).

Similarly, there are multiple lines of evidence that DNA damage promotes heart disease. Oxidative DNA adducts are elevated in the myocardium and serum of patients with heart failure (Kono et al., 2006; Zhang et al., 2017). Radiation therapy for thoracic tumors, including breast cancer, lung cancer, and Hodgkin lymphoma, is associated with increased incidence of cardiovascular disease (Swain et al., 2003). Furthermore, cancer patients treated with genotoxic agents such as anthracyclines or radiation therapy are at increased risk of cardiovascular disease, most commonly left ventricular dysfunction and cardiomyopathy (Higgins et al., 2015). In fact, 65% of patients treated with anthracyclines, for example doxorubicin, develop left ventricular dysfunction (Lipshultz et al., 1991, 1995). Doxorubicin cardiotoxicity is dose‐dependent, with a 5% incidence of heart failure in patients with a cumulative dose of less than 400 mg/m^2^, but this increases to 48% at a cumulative dose of 700 mg/m^2^ (Nielsen et al., 2017).

Here, we demonstrate that rendering cardiac myocytes DNA repair deficient by deleting a critical subunit of the repair endonuclease ERCC1‐XPF causes, not surprisingly, profound sensitivity to genotoxic agents. This includes sensitivity to the cancer chemotherapeutic agent doxorubicin. This supports the notion that the anthracycline induces cardiotoxicity via damaging DNA in cardiac myocytes, a mechanism that is still under debate (Zhao & Zhang, 2017).

More importantly, our genetic approach reveals for the first time that *endogenous* DNA damage, produced spontaneously through normal cellular processes, impacts cardiac myocyte health. Deleting *Ercc1* in cardiac myocytes causes a profound pathologic effect in the heart, leading to dilated cardiomyopathy and sudden death in mice by 6 months of age, independent of any exogenous genotoxin (Figures 1 and 2). We demonstrated previously that even in WT, repairproficient mammals, oxidative DNA lesions are increased in some tissues of aged organisms compared to young (Wang et al., 2012), indicating that DNA repair is never 100% efficient. This suggests that spontaneous, endogenous DNA damage, which is unavoidable and never fully repaired, could contribute to the causation of heart disease in mammals. In support of our data, a very recent study found increased transcript variants (mutations) in cardiac tissue of mice in which *Ercc1* was deleted, consistent with the notion that *Ercc1* is critical for removal of endogenous DNA damage in the heart (De Majo et al., 2021).

We also provide evidence that genotoxins potently drive apoptosis of cardiac myocytes in vitro (Figures 3 and 4). Similar results are reported in the accompanying manuscript be de Boer et al. in four related but unique DNA repair‐deficient mouse strains. We also found increased apoptosis in cardiac tissue of untreated *Ckmm‐Cre* ^*+/−*^ *;Ercc1* ^*−/fl*^ mice, illustrating that endogenous DNA damage can drive apoptosis in vivo. This reveals a novel cell autonomous mechanism that may contribute to idiopathic cardiomyopathy. Consistent with unrepaired DNA damage driving apoptosis of *Ercc1*‐deficient cardiac myocytes and thinning of the heart walls, spontaneous oxidative DNA adducts are decreased in cardiac tissue of the mutant mice (Figure S15). Interestingly, while dilated cardiomyopathy is not a classic age‐related disease, it is associated with diverse syndromes that promote multi‐organ accelerated aging including cancer therapy, HIV, and diabetes (Bloom et al., 2016; Prandi et al., 2022; Tschöpe et al., 2021).

Another cell fate in response to genotoxic stress is cellular senescence (Niedernhofer et al., 2018). There was no evidence of increased senescence in cardiac tissue from the *Ckmm‐Cre* ^*+/‐*^ *;Ercc1* ^*−/fl*^ mice, in which *Ercc1* is deleted only in differentiated myocytes. In contrast to this, we reported increased expression of senescence markers in cardiac tissue of *Ercc1* ^*−*/D^ mice with systemic depletion of *Ercc1* expression, and in very old wild‐type mice (Yousefzadeh et al., 2020). This could indicate that cell types in the heart other than terminally differentiated cardiomyocytes senesce with accelerated or natural aging, for example, fibroblasts, endothelial cells, and tissue‐resident immune cells. Indeed, there is ample evidence that each of these cell types does senescence readily from many laboratories and in a variety of model systems. There is convincing data supporting detection of senescence in cardiomyocytes isolated from adult mouse heart (Anderson et al., 2019). Differences between the two studies that might contribute to disparate outcomes include the genetic background, age, state of health, and inducer of senescence. Spatial detection of senescent cells in tissue sections, in combination with stringent cell lineage markers, will need to be applied under numerous conditions (e.g., animal age, sex, and perturbations/stress paradigms) to define what cell types senesce in vivo.

In vivo, cardiac myocytes appear substantially more sensitive to genotoxic stress than skeletal muscle myocytes. We show that in *Ckmm‐Cre* ^*+/−*^ *;Ercc1* ^*−/fl*^ mice, *Ercc1* is deleted in striated muscle of both organs, yet the mice did not show a phenotype in skeletal muscle (Figure S6) up to their time of death at 6–7 months of age (Figure 1). This may reflect the fact that cardiac myocytes are under constant metabolic demands, which could create more reactive species and thereby cause more DNA damage in cardiac myocytes than occurs in skeletal myocytes.

It is well‐established that DNA damage activates the guardian of the genome, p53 (Williams & Schumacher, 2016). Indeed, we found increased levels of p53 protein, phospho p53, and p21 a downstream target of p53, in cardiac tissue of mice in which DNA repair was genetically depleted (Figure 3a–c), as well as increased transcripts of p53 target genes *Bax*, *Puma*, *Noxa*, and *Apaf1* (Figure 3g–j), indicating increased p53 transcriptional activation. p53 activation in response to genotoxic stress increased the expression of antioxidant genes in cardiac tissue of DNA repair‐deficient mice, which is a well‐recognized role of p53 (Budanov, 2014).

**FIGURE 6:**
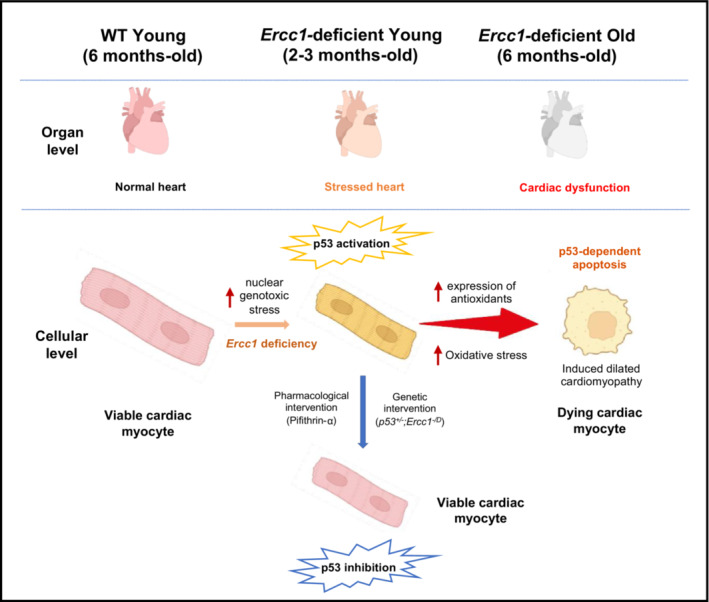
Model for the mechanism by which DNA damage drives heart failure. Unrepaired endogenous *DNA* damage leads to transcriptional activation of p53. If genotoxic stress and p53 activation occur chronically, antioxidant defenses are overcome. The direct cause of cardiac myocyte loss and ultimately cardiomyopathy in response to genotoxic stress is oxidative stress and p53‐dependent apoptosis as inhibition of either rescues cardiac myocyte viability. Figure created using bioRENDER.com (https://biorender.com/).

Chronically, this is not sufficient to attenuate oxidative stress in cardiac tissue (Figure 5). In vitro, apoptosis of cardiac myocytes (treated with genotoxins or not) is suppressed by genetic or pharmacologic inhibition of p53 (Figure 4a–c). Similarly, overexpression of mitochondrial‐targeted catalase reduces markers of heart failure (*Anp* and *Bnp*) in DNA repair‐ deficient mice (Figure 5g,h). Thus, mechanistically, DNA damage, regardless of its source (exogenous or endogenous), is sufficient to drive cardiac myocyte apoptosis, resulting in dilated cardiomyopathy and sudden death via a mechanism that is p53‐dependent and mediated by oxidative stress (Figure 6).

p53 is known to be elevated in diseased cardiac tissue compared to healthy human tissue, as are markers of apoptosis (Song et al., 1999). This is consistent with our findings in mice and supports a causal role for endogenous DNA damage in human heart disease. Our findings with *Ercc1* deficiency are also consistent with a prior study demonstrating that deletion of *Xrcc1*, essential for repair of spontaneous, endogenous DNA singlestrand breaks, exacerbates heart failure in a mouse model of pressure overload‐induced heart failure (Higo et al., 2017). However, spontaneous disease was not detected in that mutant strain. Interestingly, genetic deletion of the DNA damage response gene *Atm* rescues the heart failure phenotype in the mice lacking XRCC1 (Higo et al., 2017), indicating that it is the response to genotoxic stress that drives heart disease, analogous to our results upon inhibition of p53. Together, our study and the accompanying study by de Boer et al. provide compelling evidence that cardiac myocytes are exquisitely sensitive to endogenous DNA damage, which drives p53‐mediated apoptosis of damaged cells. This sheds light on opportunities for therapeutic interventions such as radical scavengers to prevent endogenous DNA damage and inhibitors of the DNA damage response to prevent loss of irreplaceable post‐mitotic cells (Robinson et al., 2018).

## 4 EXPERIMENTAL PROCEDURES

Detailed methods are provided as Supporting Information.

### 4.1 Animal welfare

All animal experiments were approved by the Scripps Research Institute, or the University of Minnesota Institutional Animal Care and Use Committees and performed in accordance with NIH Guide for Care and Use of Laboratory Animals. Mice were euthanized by CO_2_ inhalation followed by cervical dislocation as a secondary means of death in accordance with the American Veterinary Medical Association Guidelines for the Euthanasia of Animals. Anesthesia was delivered in an induction chamber or facemask using isoflurane (3%–4% for induction and 1%–2% for maintenance). Mice were monitored for anesthetic depth, respiratory rate and function, body temperature, and heart rate during anesthesia and provided ophthalmic ointment. Following anesthesia, mice were placed into cages on warming pads to provide thermal support and monitored for recovery. Fluid support was provided on an as needed basis.

### 4.2 Mice

Ckmm‐Cre mice (obtained from The Jackson Laboratory, strain name B6.FVB(129S4)Tg(Ckmm‐cre)5Khn/J) were described previously (Wai et al., 2015). The null allele of *Ercc1* was constructed by inserting a neomycin cassette into exon 7 of the murine locus (Weeda et al., 1997). The floxed allele of *Ercc1* was constructed by inserting the cDNA for exons 7–10 of *Ercc1* along with a neomycin cassette all flanked by *loxP* sites into the *Ercc1* locus in intron 6 (Figure S1a) (Yousefzadeh et al., 2021). *Ercc1* ^*+/fl*^ FVB/n were crossed with *Ercc1* ^*+/−*^ *;Ckmm‐Cre* ^*+/−*^ C57BL/6 mice to create *Ckmm‐Cre* ^*+/−*^ *;Ercc1* ^*−/fl*^ mice (Figure S1b) carrying one knockout and one floxed allele excised by Cre recombinase only in differentiated myocytes. *Ercc1* ^*+/fl*^ FVB/n were crossed with mitCAT;*Ckmm‐Cre* ^*+/−*^;*Ercc1* ^*+/−*^ C57BL/6 mice to create mitCAT;*CkmmCre* ^*+/−*^ *;Ercc1* ^*−/fl*^ mice (Figure S1c) (Schriner et al., 2005). All experimental animals produced in an f1 genetic background of 50% FVB/n and 50% C57BL/6 created by breeding fully inbred mice of two different backgrounds.

## AUTHOR CONTRIBUTIONS

Sara J. McGowan, Ryan D. O'Kelly, Luise A. Angelini and Danielle Hennessy‐Wack did the animal experiments. Chastity L. Healy and Timothy D. O'Connell performed and analyzed the ECHO data. Chastity L. Healy, Rajesh Vyas, Chathurika Henpita, and Tra L. Kieu did the cardiac myocyte isolation and experiments. Aiping Lu, Mitra Lavasani, and Johnny Huard characterized the skeletal muscle. Yuxiang Cui and Yinsheng Wang measured cyclopurine adducts. Eric E. Kelley did the redox‐related experiments and performed the ascorbate free radical EPR measurements. Mark A. Ross, Donna Beer‐Stolz, Claudette M. St. Croix, and Simon C. Watkins did tissue staining and imaging. Smitha P.S. Pillai and Warren Ladiges did the histopathological analysis. Aditi U. Gurkar, Rajesh Vyas, Chathurika Henpita, Matthew J. Yousefzadeh, Tra L. Kieu, and Sanjay Chandrasekhar contributed to molecular analysis. Rajesh Vyas, Chathurika Henpita, and Tra L. Kieu did the studies with p53 and mitCAT mouse tissues. Rebecca R. Vanderpool, Timothy N. Bachman, Charles McTierman, Ana L. Mora, and Aditi U. Gurkar contributed to the original cardiac function characterization of the mice. Aditi U. Gurkar, Rajesh Vyas, Chathurika Henpita, Timothy D. O'Connell, Paul D. Robbins, and Laura J. Niedernhofer wrote the manuscript with the help of all authors. Laura J. Niedernhofer and Timothy D. O'Connell supervised the study.

## FUNDING INFORMATION

Funding for this project came from the NIH: P01 AG043376 (PDR, LJN, YW, EEK, DBS, CMS, SCW, WLL), K99‐R00 AG049126 (AUG), R56 AG059676, R01 AG063543, P01 AG062413 (PDR, LJN), P20 GM109098 and AHA 19TPA34850089 (EEK), R24 AG047115 (WLL) and P01 HL103455‐06 (ALM), NSF 1359369 (SC), Irene Diamond Fund/AFAR Postdoctoral Transition Award (MJY), The Institute for Transfusion Medicine and The Hemostasis and Vascular Biology Research Institute phenotyping core (ALM). R01 HL130099 and R01 HL152215 (TDO). The Jabilain Family Foundation provided philanthropic support for research in cardiology to Scripps Research Institute, which supported this project.

## CONFLICT OF INTEREST STATEMENT

LJN and PDR are co‐founders of Itasca Therapeutics, a start‐up company developing novel senolytics.

## Supporting information


Video S1

Video S2

Figure S1

Table S1

## Data Availability

All data from this study are available in Supporting Information.
