# Supplementary material for: Loss of DNA repair mechanisms in cardiac myocytes induce dilated cardiomyopathy
Source: Aging Cell. 2023 Feb 3;22(4):e13782. doi: 10.1111/acel.13782 (PMC10086531; doi:10.1111/acel.13782)
Supplement: Supplementary file 3 — Figure S1 [file ACEL-22-e13782-s004.pdf]

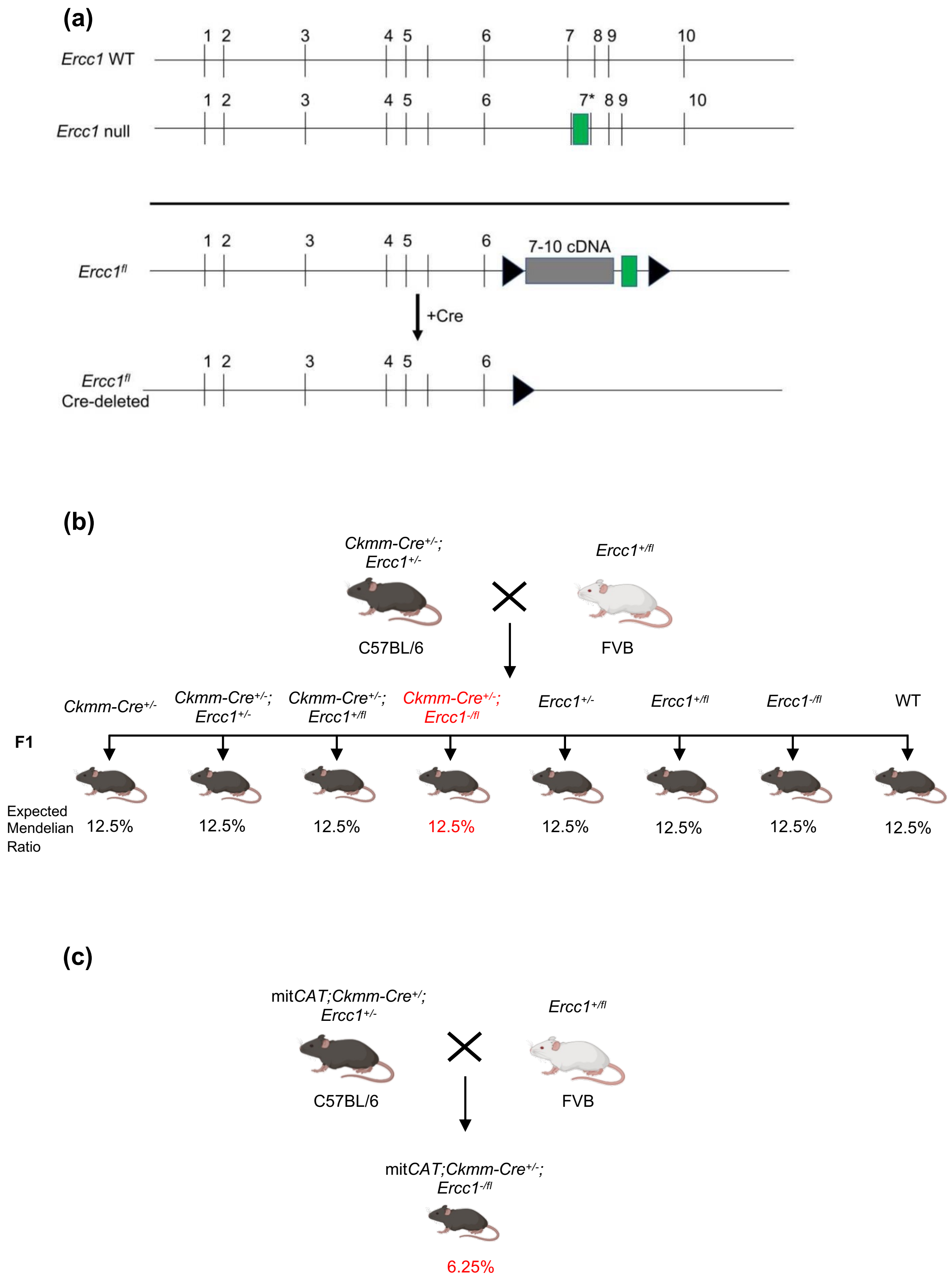

Supplementary Figure 1: Strategy to knock-out *Ercc1* expression in striated muscle of mice. (a). Schematic representation of the wild-type murine *Ercc1* locus, as well as the knock-out, floxed, and recombined alleles. **(b).** Illustration of breeding scheme for *Ckmm-Cre*$^{+/-}$;*Ercc1*$^{-/fl}$ and **(c).** *mitCAT;Ckmm-Cre*$^{+/-}$;*Ercc1*$^{-/fl}$ mice. See Supplementary Table 1 for frequency of live births. Figure created using bioRENDER.com (https://biorender.com/).

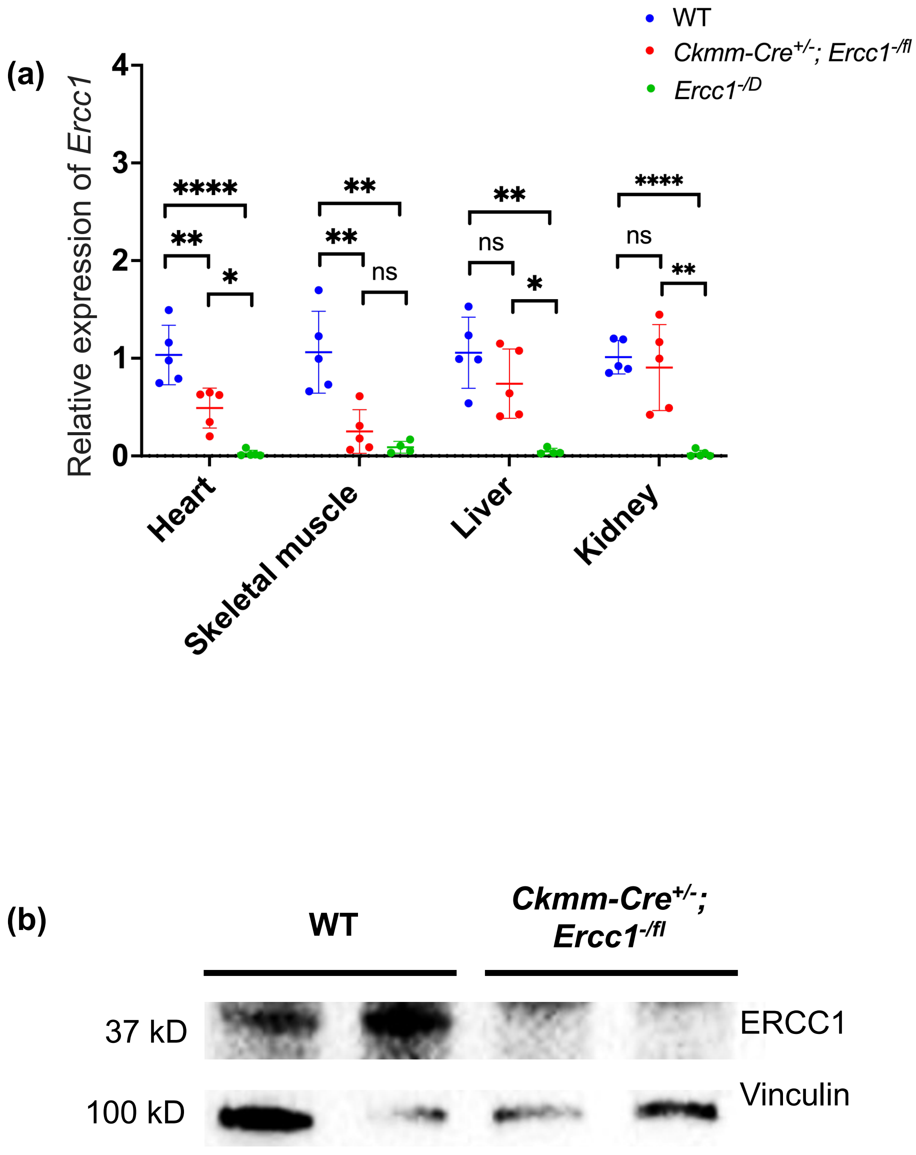

Supplementary Figure 2: *Ercc1* expression in heart, skeletal muscle, liver and kidney from the tissue specific-mutant *Ckmm-Cre*$^{+/-}$*;Ercc1*$^{-/fl}$ mice, *Ercc1*$^{-/D}$, and wild-type (WT) animals. (a). *Ercc1* mRNA levels as measured by qRT-PCR. One-way ANOVA with Tukey's multiple comparison test**,** *$p<0.05$, **$p<0.01$, ****$p<0.0001$, and ns = non-significant. See Supplementary Data Table 4 and 5 for primers and the expression of target genes respectively. **(b).** Immunoblot detection of ERCC1 protein in cardiac tissue of *Ckmm-Cre*$^{+/-}$*;Ercc1*$^{-/fl}$ mice and a littermate WT control. Vinculin was used as a loading control. See Supplementary Data Table 5 for expression of target genes.

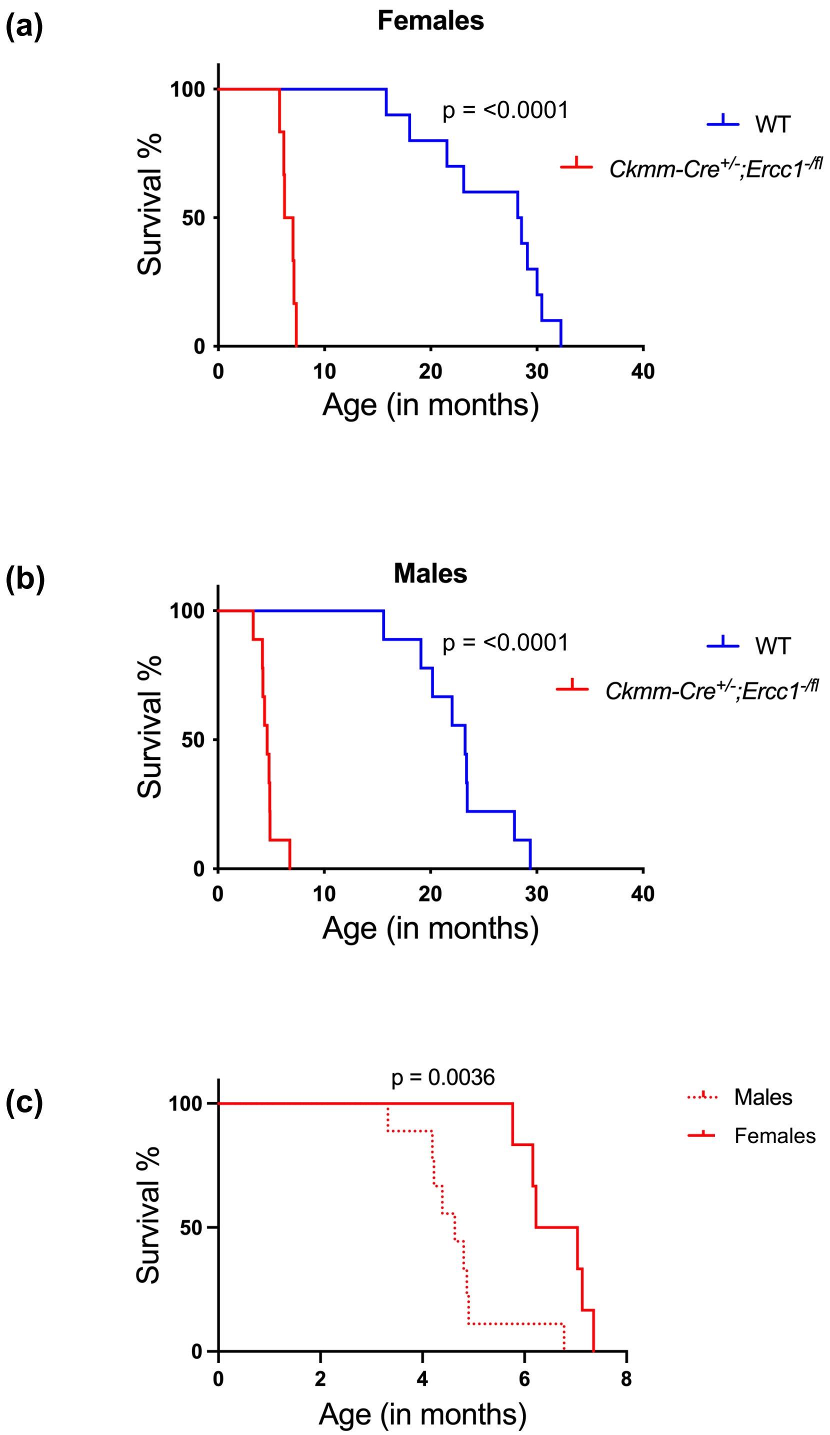

Supplementary Figure 3: Reduced lifespan in both female and male *Ckmm-Cre$^{+/-}$;Ercc1$^{-/fl}$* mice. Kaplan-Meier survival curves demonstrating that loss of *Ercc1* expression in differentiated myocytes leads to a significantly reduced lifespan of *Ckmm-Cre$^{+/-}$;Ercc1$^{-/fl}$* mice **(a).** in females (n=6-10) and **(b).** males (n=9), p<0.0001, Log- rank (Mantel-Cox) test. **(c).** Kaplan-Meier survival curves showing that loss of *Ercc1* in differentiated myocytes leads to a significantly reduced lifespan in male mice compared to females.

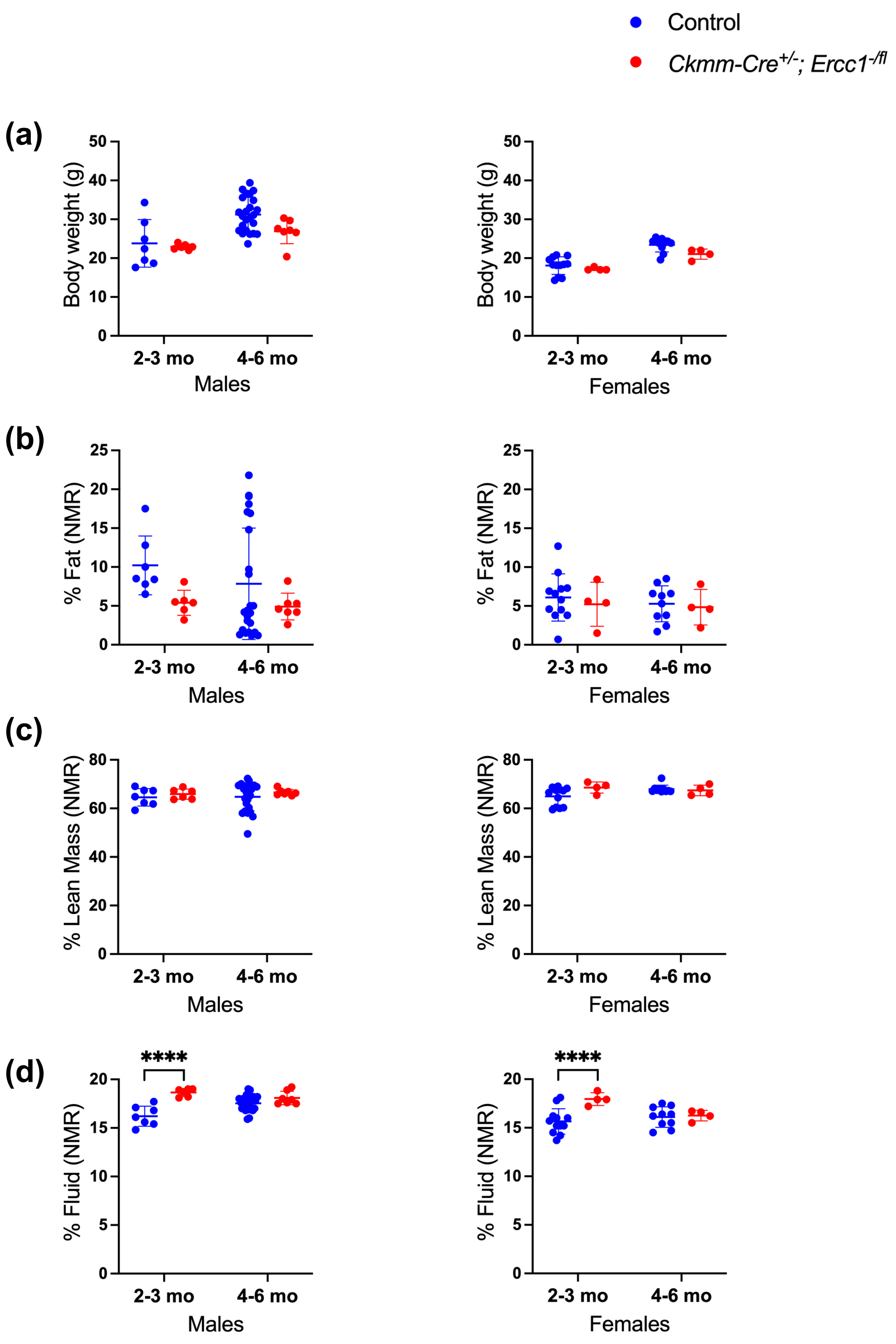

Supplementary Figure 4: Body weight and composition. (a). Weights of male and female mice at two ages. **(b-d).** Time domain nuclear magnetic resonance (NMR) measure of **(b).** body fat, **(c).** lean mass, and **(d).** fluid of 2-3- and 4-6-month-old male (left) and female (right) mice of *Ckmm-Cre*$^{+/-}$;*Ercc1*$^{-/fl}$ and WT littermates. Data are represented as the mean ± SD, n=6-24 per group, One-way ANOVA ****p<0.0001.

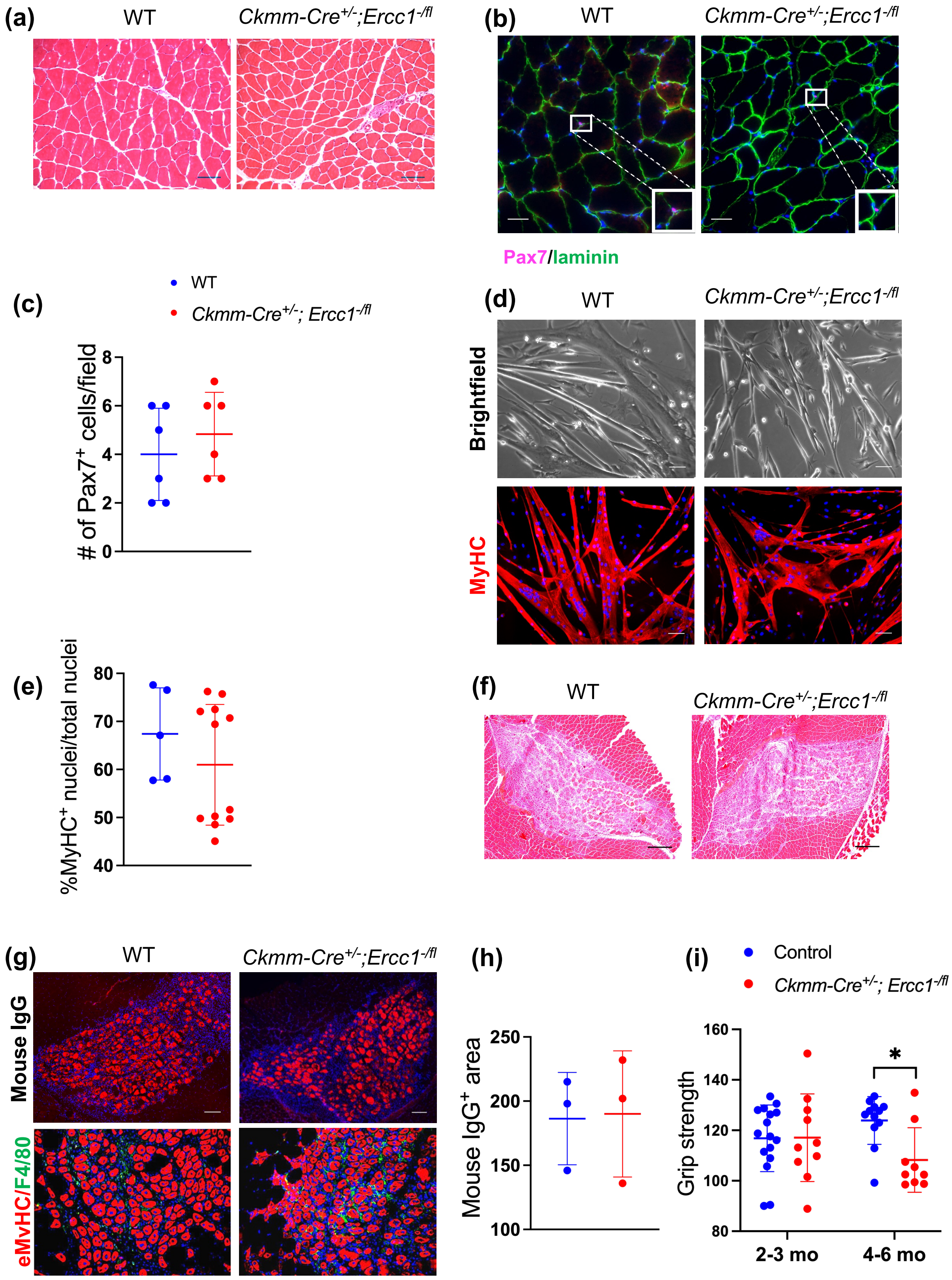

(a)
WT
Ckmm-Cre+/-;Ercc1-/fl
(b)
WT
Ckmm-Cre+/-;Ercc1-/fl
Pax7/laminin
(c)
WT
Ckmm-Cre+/-; Ercc1-/fl
# of Pax7+ cells/field
0
2
4
6
8
(d)
WT
Ckmm-Cre+/-;Ercc1-/fl
Brightfield
MyHC
(e)
%MyHC+ nuclei/total nuclei
40
50
60
70
80
(f)
WT
Ckmm-Cre+/-;Ercc1-/fl
(g)
WT
Ckmm-Cre+/-;Ercc1-/fl
Mouse IgG
eMyHC/F4/80
(h)
Mouse IgG+ area
100
150
200
250
(i)
Control
Ckmm-Cre+/-; Ercc1-/fl
Grip strength
80
100
120
140
160
*
2-3 mo
4-6 mo

Supplementary Figure 5: Analysis of skeletal muscle in *Ckmm-Cre$^{+/-}$;Ercc1$^{-/fl}$* mice. (a). H&E stained sections of quadriceps from a 6-month-old *Ckmm-Cre$^{+/-}$;Ercc1$^{-/fl}$* mouse and WT littermate showing normal histology as evaluated by light microscopy. Scale bar = 50μm. **(b).** Section of gastrocnemius muscle immunostained for Pax7 (red) to identify satellite cells and laminin (green) to identify the basal lamina, where satellite cells reside. Scale bar = 25μm. **(c).** Quantification of the number of satellite cells at 4-6-months-of-age, relative to WT. Data are represented as mean ±SD, n=6, Two-tailed, unpaired Student's *t* test, no significant differences found. **(d).** Representative images of in vitro myogenic differentiation of muscle-derived stem/progenitor cells (MDSPCs) at 4-6 month of age (top). Cells were immunostained for the terminal differentiation marker myosin heavy chain (f-MyHC in red). Scale bar = 50μm. Differentiation of MDSPCs isolated from mutant mice was not affected (n=3 cell populations from 3 mice of each genotype), nor was proliferation (data not shown). **(e).** Quantification of myogenic differentiation (number of nuclei in MyHC^{+} myotubes relative to total number of nuclei). Data represent the mean ± SD, n=5-6. **(f).** H&E stained sections of the gastrocnemius muscle to illustrate muscle regeneration 5 days after cardiotoxin injection. Numerous dark staining nuclei in the section illustrate inflammatory infiltration. No difference in muscle regeneration between mutant and WT animals was observed at 4-6 month of age (n=3). Scale bar = 100μm. **(g).** Sections of the regeneration gastrocnemius muscle immunostained for murine IgG to illustrate necrotic fibers (top) or embryonic myosin heavy chain (red) and F4/80 (green) to illustrate new regenerated myofibers and macrophages, respectively. No difference in necrosis, inflammation, or muscle regeneration after injury were observed at 4-6 months (n=3). Scale bar = 100μm. **(h).** Quantitation of mouse IgG after muscle injury. Data are represented as mean ± SD, n=3. **(i).** Grip strength of *Ckmm-Cre$^{+/-}$;Ercc1$^{-/fl}$* mice declined modestly in 4-6-month-old mice (Control genotypes$^{\#}$ n=16; *Ckmm-Cre$^{+/-}$;Ercc1$^{-/fl}$* n=9) mice per age group. Two-tailed Student's *t* test *p<.05. Data represent the mean ± SD. $^{\#}$Control group including WT, *Ercc1$^{-/fl}$*, *Ckmm-Cre$^{+/-}$* and *Ercc1$^{+/-}$* mice.

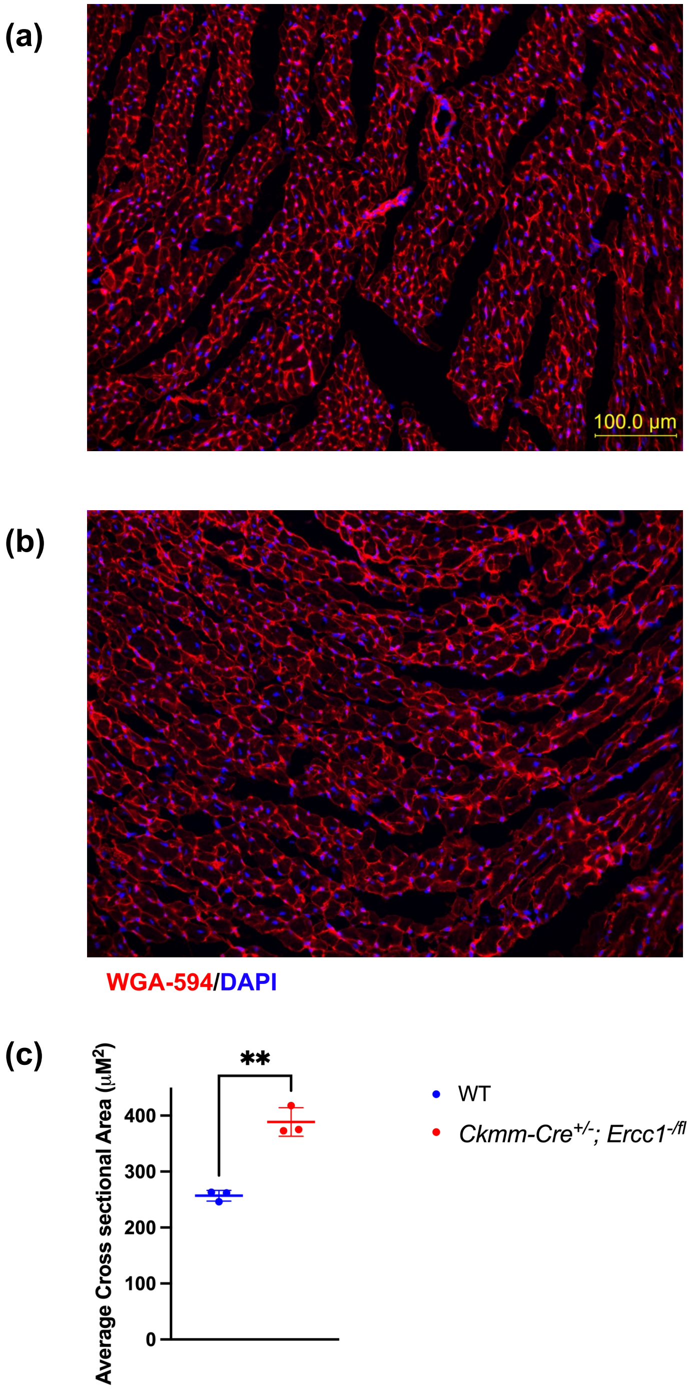

Supplementary Figure 6: Enlarged myocytes of *Ckmm-Cre$^{+/-}$;Ercc1$^{-/fl}$* Representative images of cardiac muscle tissue sections in **(a).** WT and **(b).** *Ckmm-Cre$^{+/-}$;Ercc1$^{-/fl}$* with WGA-594 staining to detect cell membrane. Sections from 5-month-old WT and *Ckmm-Cre$^{+/-}$;Ercc1$^{-/fl}$* mice (n=3). **(c).** Quantification of myocyte cross-sectional area in cardiac muscle tissue sections in WT and *Ckmm-Cre$^{+/-}$;Ercc1$^{-/fl}$* mice. Data represent the mean ± SD, n=3 per group, 7 technical replicates per animal, Two-tailed, paired Student's *t* test,**p<0.001.

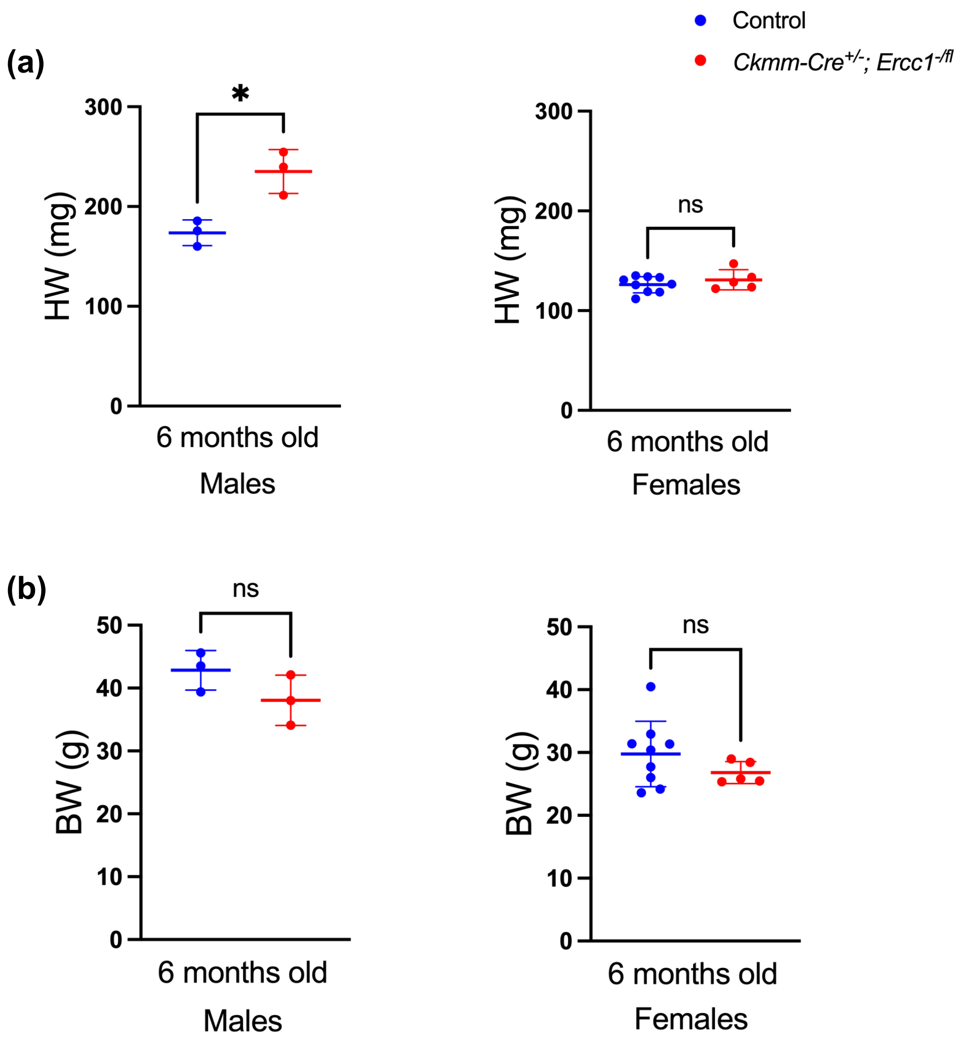

Supplementary Figure 7: Body weight (BW), and heart weight (HW) at 6-months-of-age. (a). Heart weights and **(b).** Body weight of male and female mice at 6-months-of-age. Data represent the mean ± SD, n = 3-9 mice per group. Two-tailed, unpaired Student's *t*-test, ns = non-significant.

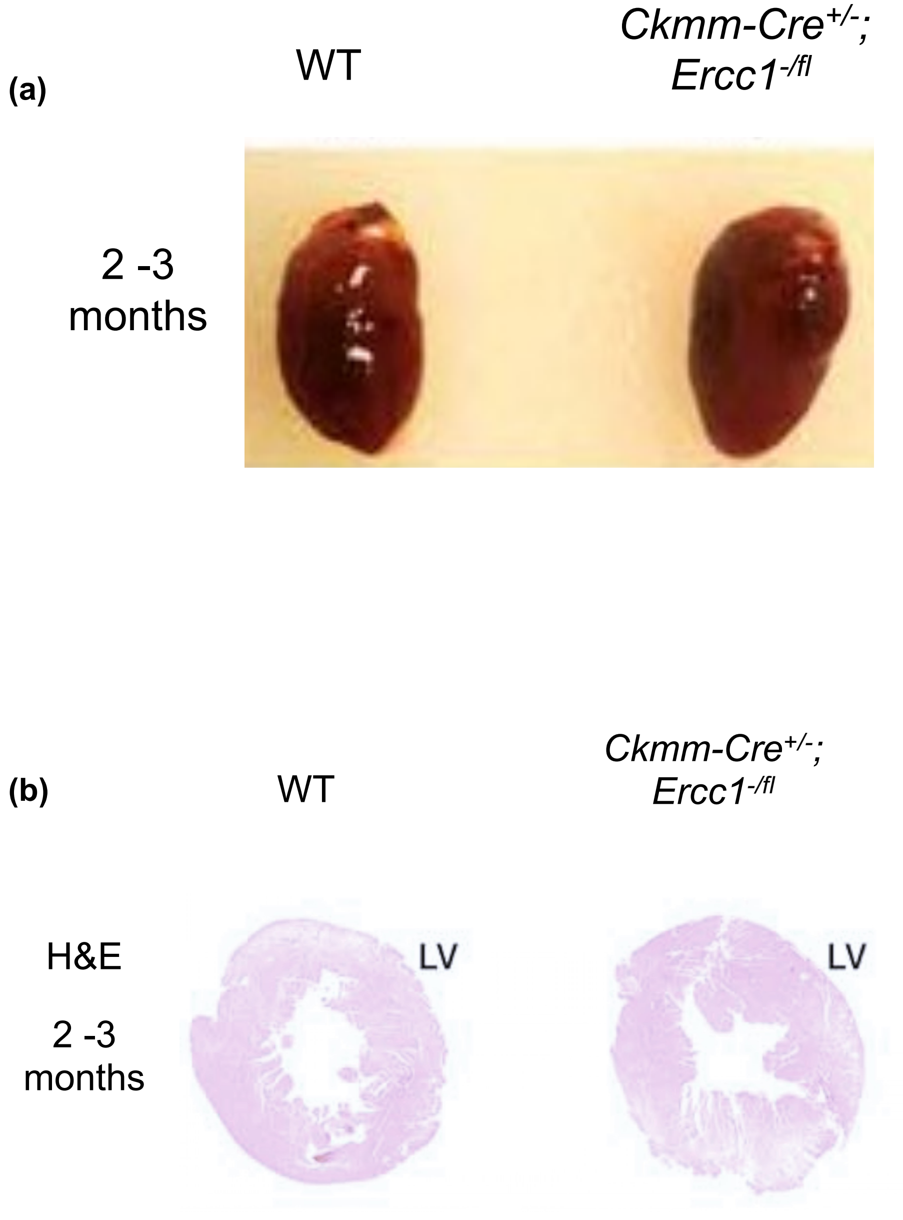

Supplementary Figure 8: Morphologically normal heart in young adult *Ckmm-Cre$^{+/-}$;Ercc1$^{-/fl}$* mice. (a). Representative images of hearts from a *Ckmm-Cre$^{+/-}$;Ercc1$^{-/fl}$* mouse and WT littermate at 2-3-months-of-age. **(b).** Representative images of H&E stained transverse section of the heart from a *Ckmm-Cre$^{+/-}$;Ercc1$^{-/fl}$* mouse and a WT littermate at 2-3-months-of-age. LV = left ventricle.

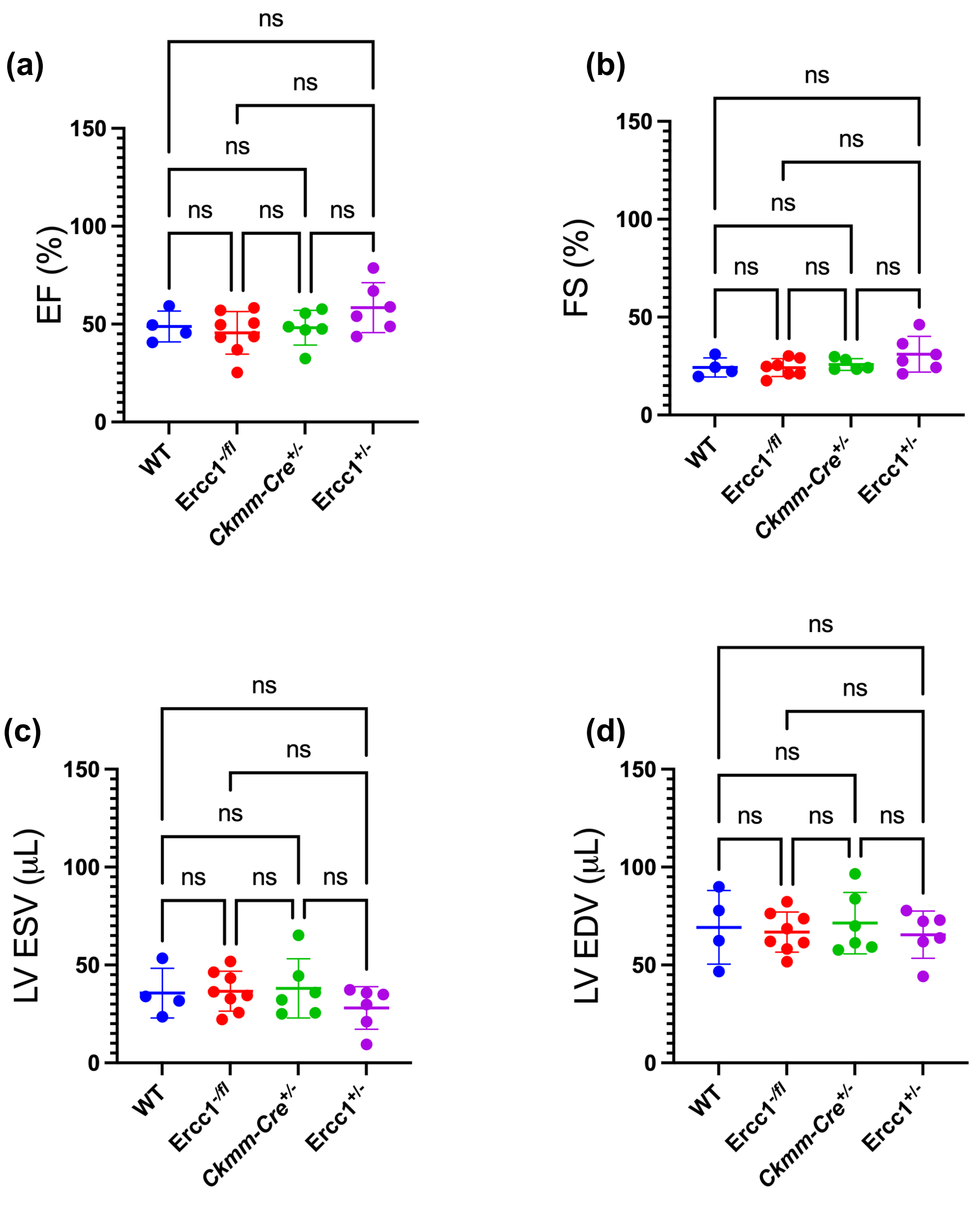

Supplementary Figure 9: Cardiac function in control animals by genotype. Echocardiography data from the control group in main Figure 2 broken out by genotype. **(a).** Ejection fraction; **(b).** fractional shortening; **(c).** left ventricular end systolic volume; **(d).** left ventricular end diastolic volume. Data represent the mean ± SD (n=4-8 per group, 6-months-of-age). One-way ANOVA with Tukey's multiple comparison test, ns = non-significant.

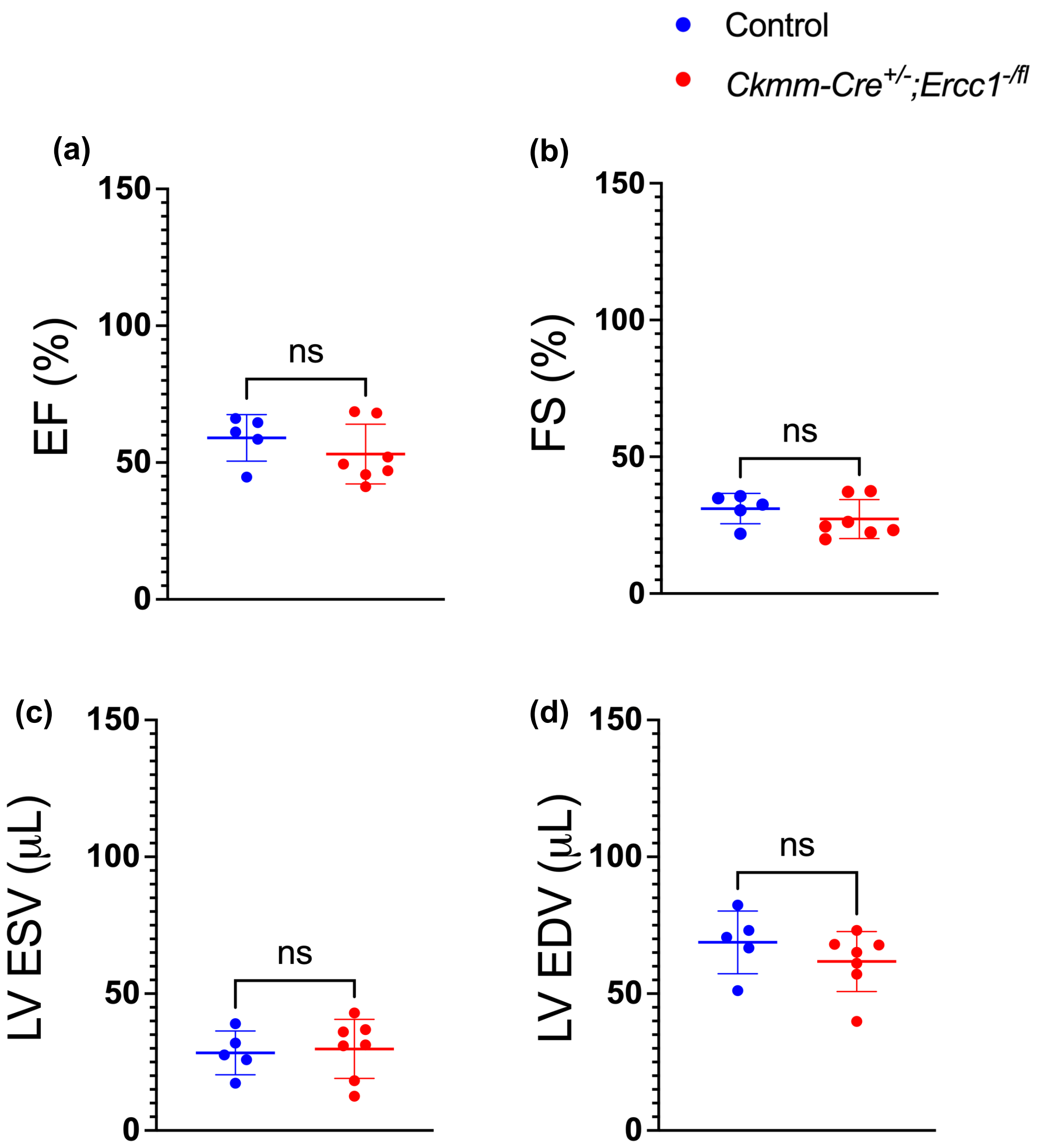

Supplementary Figure 10: Young adult *Ckmm-Cre$^{+/-}$;Ercc1$^{-/fl}$* mice do not have impaired cardiac function. Echocardiography data from 3-4-month-old mutant animals and littermate controls was used to calculate **(a).** Ejection fraction; **(b).** fractional shortening; **(c).** left ventricular end systolic volume; **(d).** left ventricular end diastolic volume. Data represent the mean ± SD, n=5-7 per group. Welch's t test (unpaired, two-tailed) ns = non-significant.

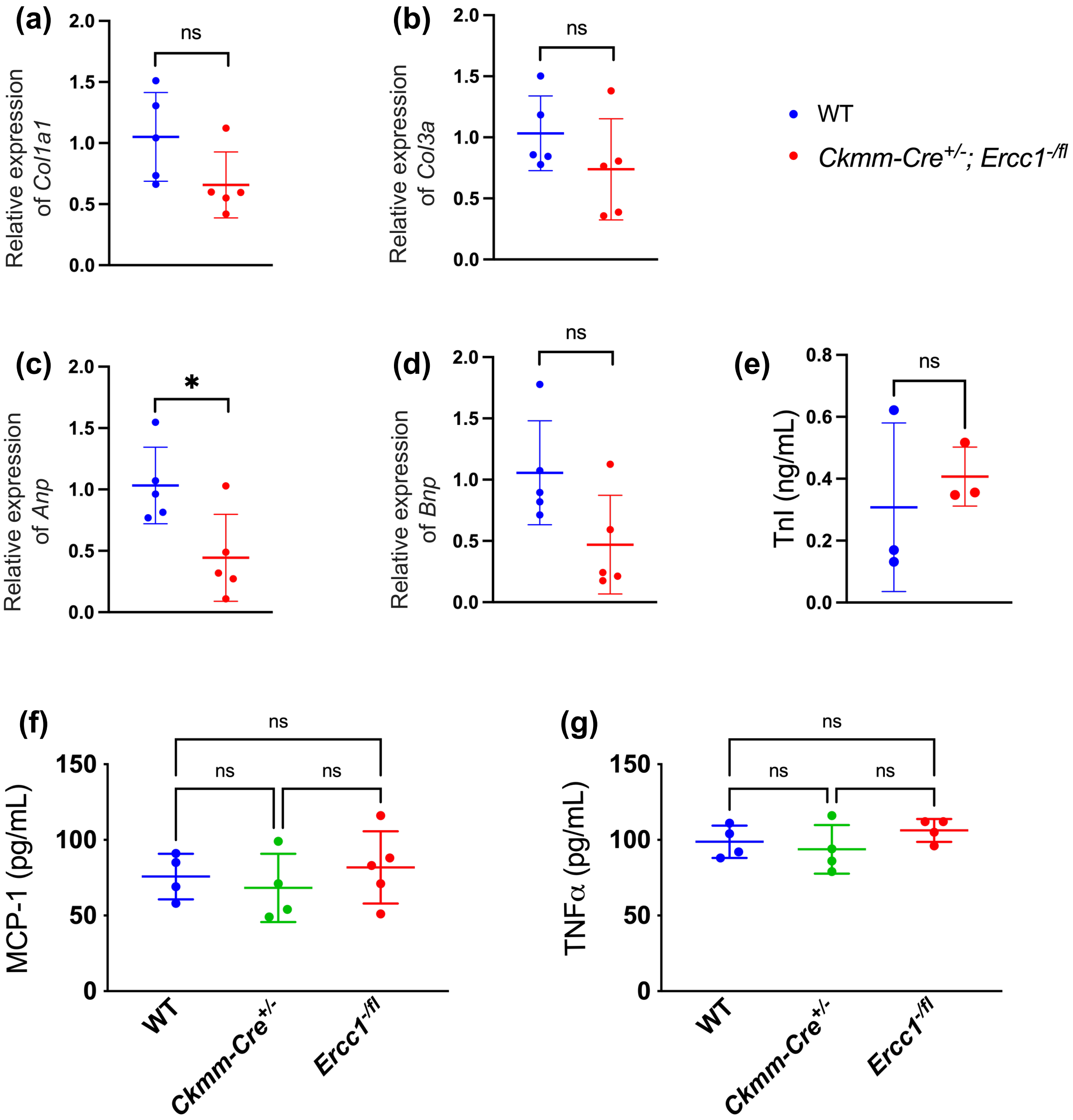

Supplementary Figure 11: Expression of collagen genes and heart failure markers in young adult mice. qRT-PCR measure of the relative expression of the following genes in left ventricular tissue from mutant and WT mice at 2-3 months of age. **(a).** *Cola1a;* **(b).** *Col3a*; **(c).** *Anp*, and **(d).** *Bnp*, as markers of cardiac fibrosis and failure. Data represent the mean ± SD, n=5 animals per genotype. See Supplementary Data Table 4 and 5 for primers and the expression of target genes respectively. **(e).** Serum cardiac troponin measured by ELISA. Data represent the mean ± SD (n = 3 mice, 2-3-months of age). Two-tailed, unpaired Student's *t* test, ns = non-significant. **(f-g).** Levels of inflammatory markers in serum of control mice by genotype. **(f).** MCP-1 and **(g).** Tumor Necrosis Factor-a (TNF-a) in mice of indicated genotypes. Data represent the mean ± SD (n=4-5 per group 2-3-months of age). One-way ANOVA with Tukey's multiple comparison test, ns = non-significant.

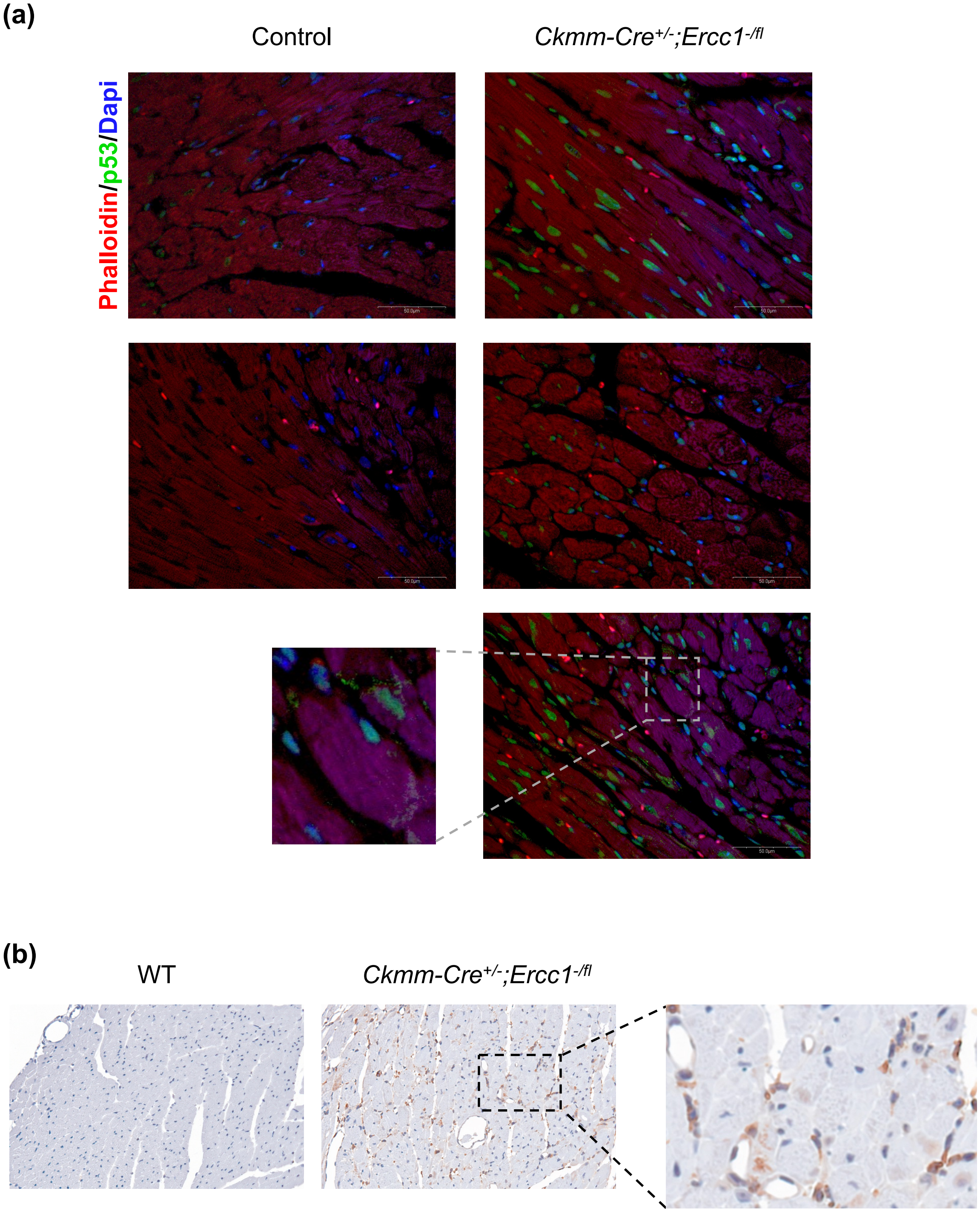

Supplementary Figure 12: Elevated levels of p53 in cardiac tissue of *Ckmm-Cre$^{+/-}$;Ercc1$^{-/fl}$* mice. (a). Representative fluorescent images of cardiac muscle tissue sections with immunodetection of p53 and staining with phalloidin to detect actin (cardiac myocyte architecture). Sections from 5-month-old control and *Ckmm-Cre$^{+/-}$;Ercc1$^{-/fl}$* mice (n=2, or 3, respectively). (**b).** Immunohistochemistry to detect p53 in cardiac tissue sections from 6-month-old WT and *Ckmm-Cre$^{+/-}$;Ercc1$^{-/fl}$* mice.

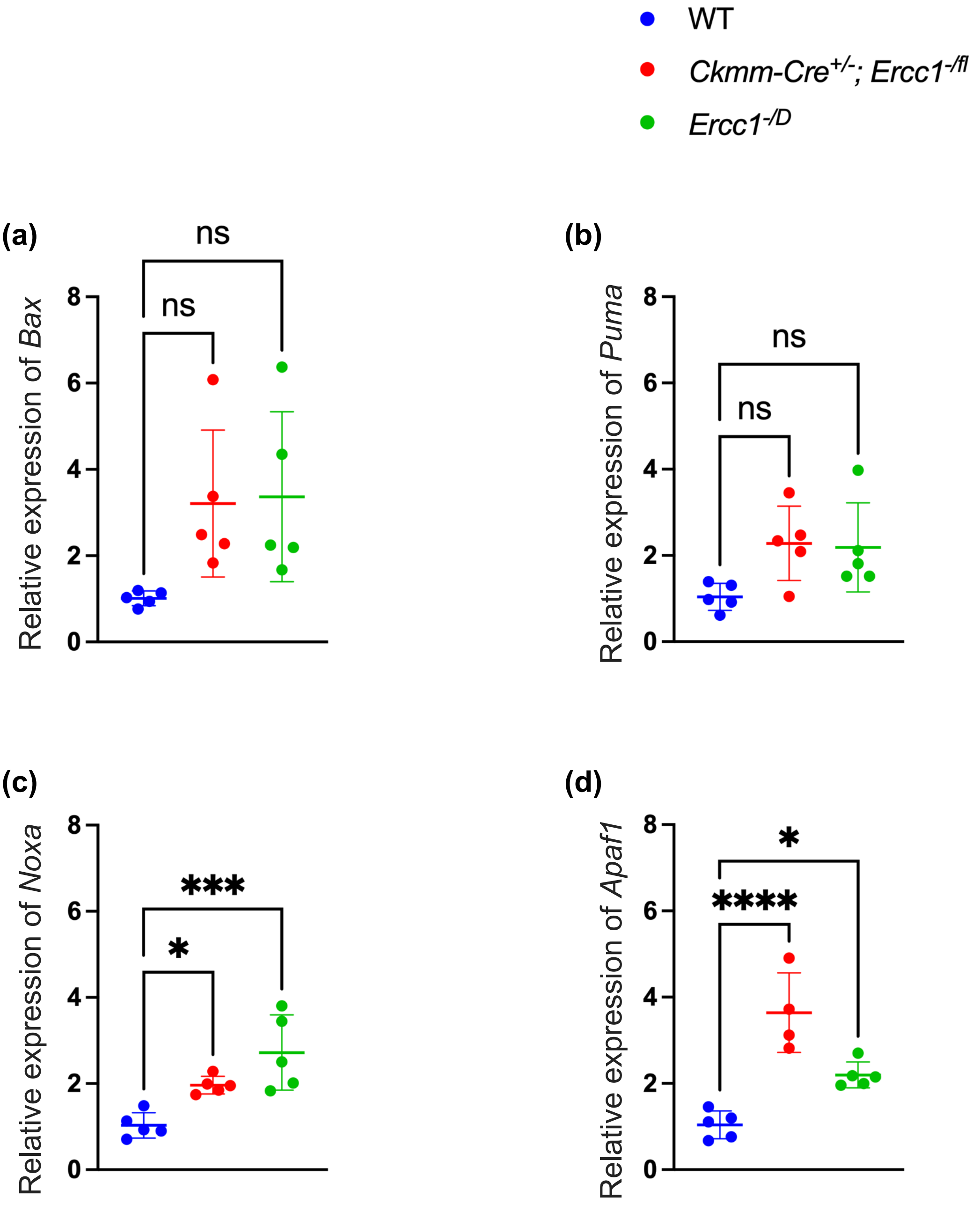

Supplementary Figure 13: Expression of pro-apoptotic p53 target genes in heart tissue of young adult mice. Expression of **(a).** *Bax*, **(b).** *Puma*, **(c).** *Noxa*, and **(d).** *Apaf1* in cardiac tissues from 2-3-month-old mice of the indicated genotypes measured by qRT-PCR. Graphed is the mean ± SD. n=4-5 animals per genotype. One-way ANOVA was used for statistical analysis, *p<0.05; ***p<0.001; ****p<0.0001 and ns = non-significant. See Supplementary Data Table 4 and 5 for primers and the expression of target genes respectively.

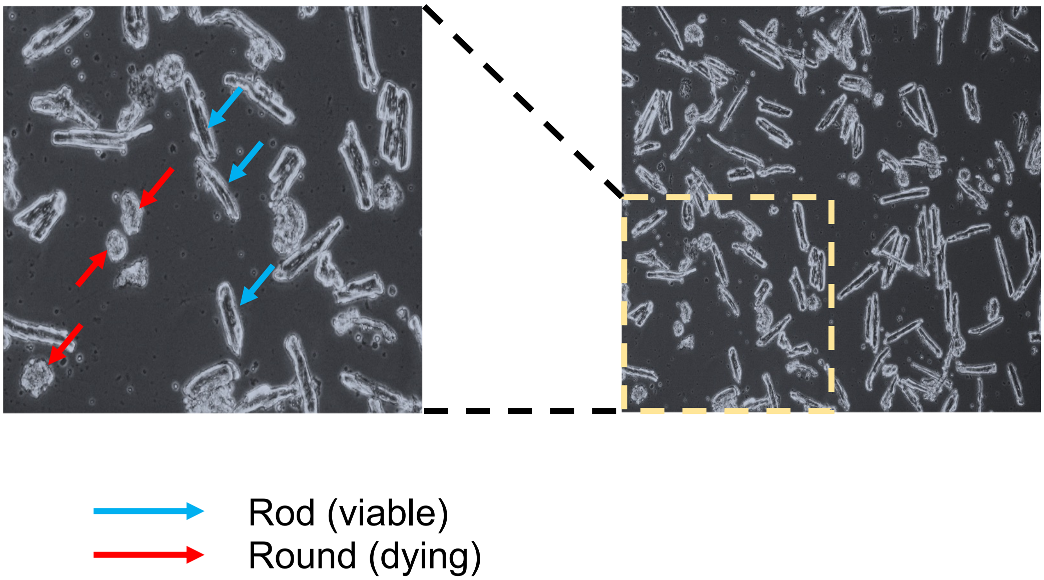

Supplementary Figure 14: Morphology of viable (rod) and dying (round) cardiac myocytes in vitro.

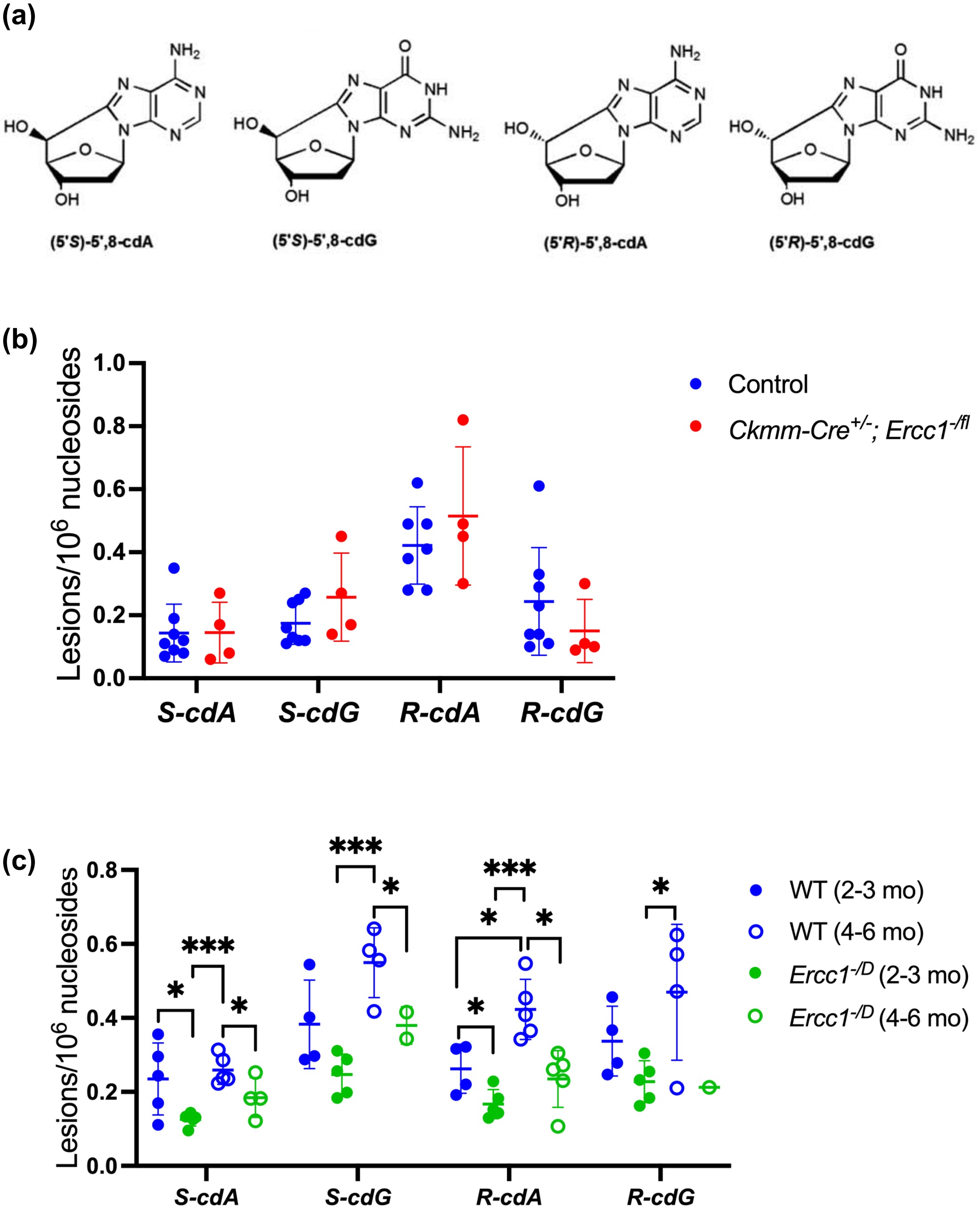

Supplementary Figure 15: Quantitation of cyclopurine oxidative DNA lesions in cardiac tissue. (a). Structures of the four cyclopurine (cPus) DNA adducts *S*-cdA, *S*-cdG*,* *R*-cdA and, *R*-cdG spontaneous oxidative DNA lesions repaired by nucleotide excision repair, an ERCC1-dependent DNA repair mechanism. **(b).** Quantitation of cPus in cardiac tissue of 4-5-month-old *Ckmm-Cre$^{+/-}$;Ercc1$^{-/fl}$* mice and control littermates. Graphed is the mean ±SD, n=4-8 animals per group. All ns by one-tailed, Student's *t* test. **(c).** Quantitation of cPus in cardiac tissue of 2-3-month-old and 4-6-month-old *Ercc1$^{-/D}$* and WT mice. Graphed is the mean ± SD. n=1-5 animals per group. One-way ANOVA *p<0.05; ***p<0.001.

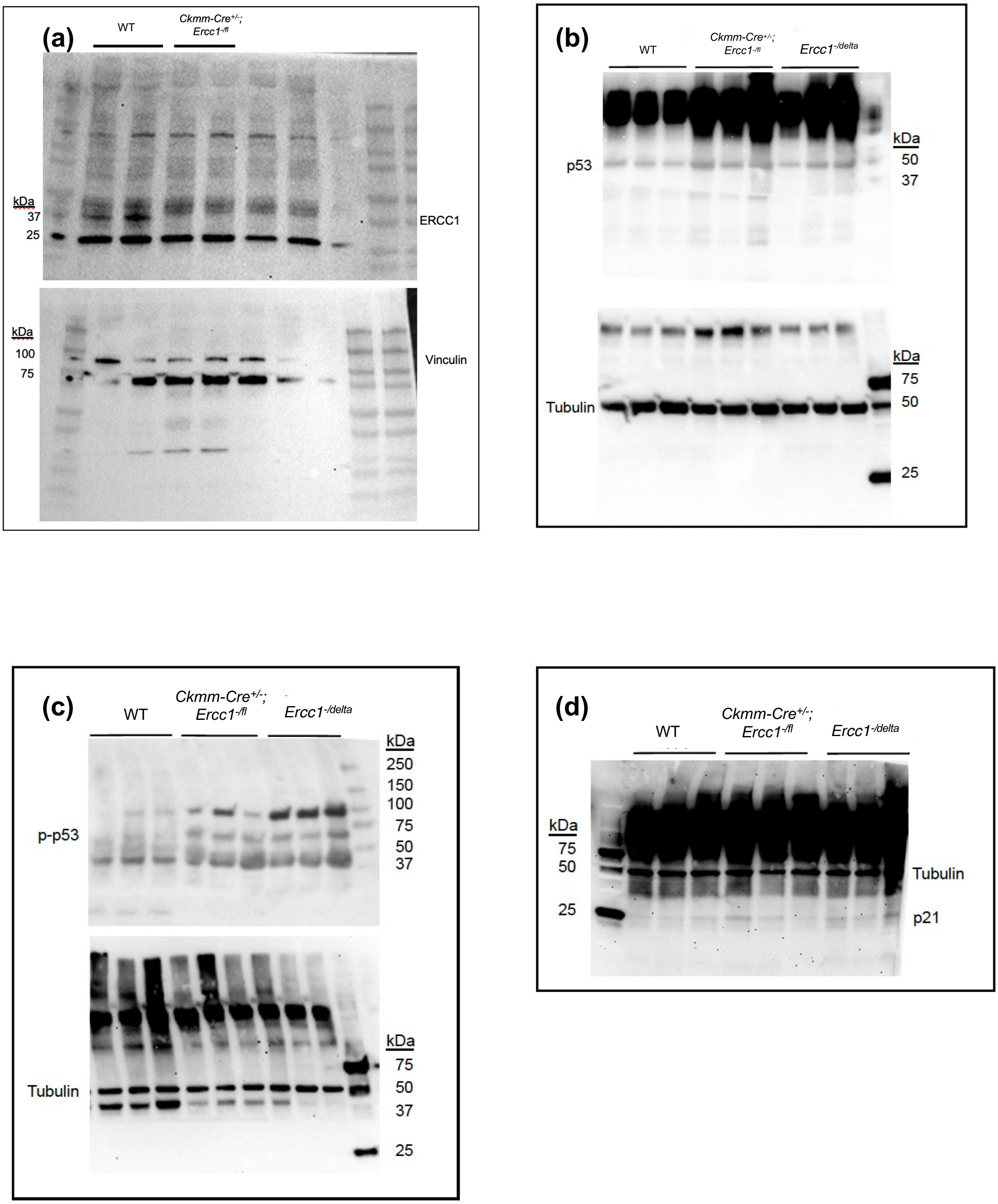

Supplementary Figure 16: Uncropped western blots with marker ladders. (a). Immunoblot detection of ERCC1 protein in tissue lysates from the heart of *Ckmm-Cre*$^{+/-}$;*Ercc1*$^{-/fl}$ mice and a littermate WT controls. Vinculin was used as a loading control. **(b-d).** Immunoblot detection of p21, p53 or (phospho) p-Ser15-p53. Tubulin was used as a loading control.
