# Supplementary material for: Loss of DNA repair mechanisms in cardiac myocytes induce dilated cardiomyopathy
Source: Aging Cell. 2023 Feb 3;22(4):e13782. doi: 10.1111/acel.13782 (PMC10086531; doi:10.1111/acel.13782)
Supplement: Supplementary file 4 — Table S1 [file ACEL-22-e13782-s001.docx]

**Supplementary Video 1 and 2: *Ckmm-Cre+/-;Ercc1-/fl* mouse is displaying dyspnea.** *Ckmm-*

*Cre+/-;Ercc1-/fl* mice at 6-month-old displayed dyspnea prior to sudden death.

**Supplementary Table 1: Frequency of live births illustrating no significant deviation from Mendelian frequency.** F (Females); M (Males)

| **Genotype** | **F** | **M** | **M + F** | **Expected Percent** |
| --- | --- | --- | --- | --- |
| ***Ckmm-Cre^+/-^*** | 13.8% | 10.7% | 12.2% | 12.5% |
| ***Ckmm-Cre^+/-^;Ercc1^+/-^*** | 11.1% | 13.2% | 12.2% | 12.5% |
| ***Ckmm-Cre^+/-^;Ercc1^+/fl^*** | 11.6% | 11.2% | 11.4% | 12.5% |
| ***Ckmm-Cre^+/-^;Ercc1^-/fl^*** | 9.5% | 10.2% | 9.8% | 12.5% |
| ***Ercc1^-/fl^*** | 9.0% | 12.2% | 10.6% | 12.5% |
| ***Ercc1^+/-^*** | 13.8% | 13.7% | 13.7% | 12.5% |
| ***Ercc1^+/fl^*** | 15.9% | 15.7% | 15.8% | 12.5% |
| **WT** | 15.3% | 13.2% | 14.2% | 12.5% |
| **Chi-square** | 7.021 | 3.731 | 8.487 |  |
| **DF** | 7 | 7 | 7 |  |
| **P value (two-tailed)** | 0.4267 | 0.8102 | 0.2916 |  |

**Supplementary Table 2: Cardiac function at 6 months of age measured by echocardiography in both sexes.**

| **Cardiac Function** | **Males** | | **Females** | |
| --- | --- | --- | --- | --- |
|  | **Control** | ***Ckmm-Cre^+/-^;Ercc1^-/fl^*** | **Control** | ***Ckmm-Cre^+/-^;Ercc1^-/fl^*** |
| EF (%) | 49.3 ± 8.07 | 23.6 ± 14.13 | 50.2 ± 13.29 | 32.8 ± 6.46 |
| FS (%) | 24.8 ± 4.84 | 11.1 ± 7.17 | 27.7 ± 7.42 | 15.3 ± 3.35 |
| ESV (μL) | 38.46 ± 10.44 | 78.17 ± 26.93 | 32.17 ± 13.04 | 45.38 ± 6.67 |
| EDV (μL) | 74.78 ± 10.27 | 99.70 ± 18.00 | 62.98 ± 12.92 | 67.49 ± 7.50 |
| HR (bpm) | 473.2 ± 49.60 | 510.7 ± 26.80 | 457.3 ± 45.87 | 506.1 ± 47.45 |
| SV (μL) | 38.22 ± 4.25 | 21.53 ± 9.23 | 30.81 ± 7.17 | 22.10 ± 4.77 |
| CO (μL/min) | 18.1 ± 2.89 | 11.0 ± 4.53 | 14.1 ± 3.70 | 11.1 ± 2.37 |
| IVS;s (mm) | 1.5 ± 0.12 | 1.1 ± 0.32 | 1.3 ± 0.15 | 1.1 ± 0.15 |
| IVS;d (mm) | 1.13 ± 0.11 | 0.98 ± 0.21 | 0.91 ± 0.13 | 0.89 ± 0.14 |
| LVID;s | 3.1 ± 0.31 | 4.1 ± 0.69 | 2.9 ± 0.48 | 3.3 ± 0.21 |
| LVID;d | 4.1 ± 0.23 | 4.6 ± 0.32 | 3.8 ± 0.31 | 3.9 ± 0.18 |
| LVPW;s (mm) | 1.30 ± 0.20 | 1.00 ± 0.20 | 1.03 ± 0.20 | 1.05 ± 0.17 |
| LVPW;d (mm) | 1.08 ± 0.19 | 0.92 ± 0.19 | 0.81 ± 0.12 | 0.93 ± 0.18 |
| E/A | 1.52 ± 0.56 | 1.66 ± 0.12 | 1.87 ± 0.31 | 2.21 ± 0.88 |
| E (mm/s) | 507.27 ± 85.97 | 435.39 ± 129.04 | 525.77 ± 68.65 | 529.79 ± 126.59 |
| E/E' | -30.81 ± 15.58 | -27.54 ± 15.48 | -21.17 ± 4.59 | -26.60 ± 5.71 |
| AoV (mm/s) | 361.82 ± 111.80 | 284.73 ± 5.37 | 287.26 ± 57.46 | 275.12 ± 239.86 |
| LVESD (mm) | 3.07 ± 0.32 | 4.14 ± 0.60 | 2.85 ± 0.47 | 3.33 ± 0.20 |
| LVEDD (mm) | 4.13 ± 0.24 | 4.63 ± 0.40 | 3.81 ± 0.31 | 3.93 ± 0.20 |

*μL = microliter; bpm = beats per minute; mm = millimeter; s = systolic; d = diastolic; EF = ejection fraction; FS = fractional shortening; EDV = end diastolic volume; ESV = end systolic volume; HR = heart rate; SV = stroke volume; CO = cardiac output; IVS = interventricular septum thickness; LVID = left ventricular internal diameter; LVPW = left ventricular posterior wall thickness; E/A = the ratio of the E to A wave – a measure of peak velocity blood flow; E = E wave velocity – the peak velocity blood flow from the LV relaxation in early diastole; E/E’ = the ratio between early mitral inflow and mitral annular early diastolic velocities; AoV = aortic velocity; LVESD = left ventricular end systolic diameter; LVEDD = left ventricular end diastolic diameter

**Supplementary Table 3: The viable cardiac myocyte counts in each treatment group before and after the treatment.**

| **Genotype** | **Treatment** | **Biological replicate** | **Pre-treatment rod count** | **Post-treatment rod count** | **% Rod Survival** |
| --- | --- | --- | --- | --- | --- |
| WT | NT | 1 | 24398 | 16154 | 66.21 |
|  |  | 2 | 14417 | 9075 | 62.95 |
|  |  | 3 | 17744 | 10572 | 59.58 |
|  | Pifithrin-⍺ | 1 | 24398 | 14990 | 61.44 |
|  |  | 2 | 17744 | 9463 | 53.33 |
|  |  | 3 | 17744 | 9353 | 52.71 |
|  | Doxo | 1 | 22180 | 8040 | 36.25 |
|  |  | 2 | 11090 | 3641 | 32.83 |
|  |  | 3 | 9981 | 2883 | 28.89 |
|  | Doxo + Pifithrin-⍺ | 1 | 26616 | 15064 | 56.60 |
|  |  | 2 | 16635 | 7874 | 47.33 |
|  |  | 3 | 16635 | 8724 | 52.44 |
|  | UV | 1 | 14417 | 5360 | 37.18 |
|  |  | 2 | 8872 | 2163 | 24.38 |
|  |  | 3 | 12199 | 3401 | 27.88 |
|  | UV + Pifithrin-⍺ | 1 | 23289 | 12901 | 55.40 |
|  |  | 2 | 13308 | 7061 | 53.06 |
|  |  | 3 | 11090 | 7560 | 68.17 |
| *Ckmm-Cre^+/-^;Ercc1^-/fl^* | NT | 1 | 15526 | 9667 | 62.26 |
|  |  | 2 | 17744 | 10683 | 60.21 |
|  |  | 3 | 14417 | 5822 | 40.38 |
|  | Pifithrin-⍺ | 1 | 15526 | 6081 | 39.17 |
|  |  | 2 | 17744 | 8983 | 50.63 |
|  |  | 3 | 14417 | 6321 | 43.85 |
|  | Doxo | 1 | 21071 | 5804 | 27.54 |
|  |  | 2 | 15526 | 3419 | 22.02 |
|  |  | 3 | 15526 | 3808 | 24.52 |
|  | Doxo + Pifithrin-⍺ | 1 | 14417 | 7042 | 48.85 |
|  |  | 2 | 16635 | 7393 | 44.44 |
|  |  | 3 | 13308 | 6820 | 51.25 |
|  | UV | 1 | 18853 | 6358 | 33.73 |
|  |  | 2 | 19962 | 6303 | 31.57 |
|  |  | 3 | 6654 | 2070 | 31.11 |
|  | UV + Pifithrin-⍺ | 1 | 16635 | 8521 | 51.22 |
|  |  | 2 | 19962 | 11645 | 58.33 |
|  |  | 3 | 6654 | 3235 | 48.61 |

**Supplementary Table 4: List of primers used to measure gene expression by qRT-PCR.**

| **Gene** | **Forward Primer (5' - 3')** | **Reverse Primer (5' - 3')** |
| --- | --- | --- |
| ***Ercc1*** | GGATTCCGTGACGATCTGAA | ACCCACCAGGAGGAAGTTTG |
| ***Cola1*** | GAGCGGAGAGTACTGGATCG | GTTCGGGCTGATGTACCAGT |
| ***Col3a*** | AGGCTGAAGGAAACAGCAAA | TAGTCTCATTGCCTTGCGTG |
| ***Anp*** | CGGAGCCTACGAAGATCCAG | TTCGGTACCGGAAGCTGTTG |
| ***Bnp*** | GCAGCTGCATCTTGAATTGCT | GGAGAACACGGCATCATTGC |
| ***Bax*** | TGAAGACAGGGGCCTTTTTG | AATTCGCCGGAGACACTCG |
| ***Puma*** | GCGGCGGAGACAAGAAGA | AGTCCCATGAAGAGATTGTACATGAC |
| ***Noxa*** | GCAGAGCTACCACCTGAGTTC | CTTTTGCGACTTCCCAGGCA |
| ***Apaf1*** | TCTGATGCTGCGCAAACAC | CACCCAAGGATCCCAAACAT |
| ***Cat*** | CTCGCAGAGACCTGATGTCC | GACCCCGCGGTCATGATATT |
| ***Hmox1*** | GAGCAGAACCAGCCTGAACT | AAATCCTGGGGCATGCTGTC |
| ***Nqo1*** | GGTAGCGGCTCCATGTACTC | CGCAGGATGCCACTCTGAAT |
| ***Gapdh*** | AAGGTCATCCCAGAGCTGAA | GTGCTTCACCACCTTCTTGA |

**Supplementary Table 5: Expression of targeted genes in different tissue of mice, measured by qRT-PCR.**

| **Figure number** | **Target tissue** | **Age group (months)** | **Target gene** | **Genotype** | **N** | **Fold gene expression** |
| --- | --- | --- | --- | --- | --- | --- |
| Main figure 1a | Heart | 2 to 3 | *Ercc1* | WT | 5 | 0.978 |
|  |  |  |  |  |  | 0.791 |
|  |  |  |  |  |  | 1.494 |
|  |  |  |  |  |  | 1.160 |
|  |  |  |  |  |  | 0.746 |
|  |  |  |  | *Ckmm-Cre^+/-^;Ercc1^-/fl^* | 5 | 0.624 |
|  |  |  |  |  |  | 0.629 |
|  |  |  |  |  |  | 0.201 |
|  |  |  |  |  |  | 0.348 |
|  |  |  |  |  |  | 0.650 |
|  | Skeletal muscle | 2 to 3 | *Ercc1* | WT | 5 | 0.661 |
|  |  |  |  |  |  | 1.697 |
|  |  |  |  |  |  | 0.994 |
|  |  |  |  |  |  | 0.731 |
|  |  |  |  |  |  | 1.227 |
|  |  |  |  | *Ckmm-Cre^+/-^;Ercc1^-/fl^* | 5 | 0.064 |
|  |  |  |  |  |  | 0.308 |
|  |  |  |  |  |  | 0.089 |
|  |  |  |  |  |  | 0.178 |
|  |  |  |  |  |  | 0.612 |
|  | Liver | 2 to 3 | *Ercc1* | WT | 5 | 1.529 |
|  |  |  |  |  |  | 0.540 |
|  |  |  |  |  |  | 0.993 |
|  |  |  |  |  |  | 1.235 |
|  |  |  |  |  |  | 0.988 |
|  |  |  |  | *Ckmm-Cre^+/-^;Ercc1^-/fl^* | 5 | 0.426 |
|  |  |  |  |  |  | 1.150 |
|  |  |  |  |  |  | 1.077 |
|  |  |  |  |  |  | 0.641 |
|  |  |  |  |  |  | 0.407 |
|  | Kidney | 2 to 3 | *Ercc1* | WT | 5 | 1.193 |
|  |  |  |  |  |  | 0.920 |
|  |  |  |  |  |  | 1.202 |
|  |  |  |  |  |  | 0.891 |
|  |  |  |  |  |  | 0.850 |
|  |  |  |  | *Ckmm-Cre^+/-^;Ercc1^-/fl^* | 5 | 0.491 |
|  |  |  |  |  |  | 1.166 |
|  |  |  |  |  |  | 0.422 |
|  |  |  |  |  |  | 1.447 |
|  |  |  |  |  |  | 0.997 |
| Main figure 2f, g | Heart LV | 5 to 6 | *Col1a1* | WT | 5 | 1.756 |
|  |  |  |  |  |  | 0.945 |
|  |  |  |  |  |  | 0.703 |
|  |  |  |  |  |  | 1.085 |
|  |  |  |  |  |  | 0.935 |
|  |  |  |  | *Ckmm-Cre^+/-^;Ercc1^-/fl^* | 5 | 1.559 |
|  |  |  |  |  |  | 3.090 |
|  |  |  |  |  |  | 5.824 |
|  |  |  |  |  |  | 2.436 |
|  |  |  |  |  |  | 7.269 |
|  |  | 5 to 6 | *Col3a* | WT | 5 | 1.501 |
|  |  |  |  |  |  | 0.741 |
|  |  |  |  |  |  | 1.255 |
|  |  |  |  |  |  | 1.085 |
|  |  |  |  |  |  | 0.728 |
|  |  |  |  | *Ckmm-Cre^+/-^;Ercc1^-/fl^* | 5 | 4.401 |
|  |  |  |  |  |  | 1.641 |
|  |  |  |  |  |  | 6.102 |
|  |  |  |  |  |  | 2.870 |
|  |  |  |  |  |  | 6.744 |
|  |  | 5 to 6 | *Anp* | WT | 5 | 0.837 |
|  |  |  |  |  |  | 1.007 |
|  |  |  |  |  |  | 1.056 |
|  |  |  |  |  |  | 1.085 |
|  |  |  |  |  |  | 1.041 |
|  |  |  |  | *Ckmm-Cre^+/-^;Ercc1^-/fl^* | 8 | 2.734 |
|  |  |  |  |  |  | 1.043 |
|  |  |  |  |  |  | 10.343 |
|  |  |  |  |  |  | 1.197 |
|  |  |  |  |  |  | 4.960 |
|  |  |  |  |  |  | 2.016 |
|  |  |  |  |  |  | 0.965 |
|  |  |  |  |  |  | 10.588 |
|  |  | 5 to 6 | *Bnp* | WT | 5 | 1.151 |
|  |  |  |  |  |  | 1.123 |
|  |  |  |  |  |  | 0.656 |
|  |  |  |  |  |  | 1.085 |
|  |  |  |  |  |  | 1.444 |
|  |  |  |  | *Ckmm-Cre^+/-^;Ercc1^-/fl^* | 5 | 5.498 |
|  |  |  |  |  |  | 2.408 |
|  |  |  |  |  |  | 4.128 |
|  |  |  |  |  |  | 6.346 |
|  |  |  |  |  |  | 6.770 |
| Main figure 3g-j | Heart | 5 to 6 | *Bax* | WT | 5 | 1.080 |
|  |  |  |  |  |  | 1.157 |
|  |  |  |  |  |  | 0.818 |
|  |  |  |  |  |  | 1.032 |
|  |  |  |  |  |  | 0.947 |
|  |  |  |  | *Ckmm-Cre^+/-^;Ercc1^-/fl^* | 5 | 15.963 |
|  |  |  |  |  |  | 50.221 |
|  |  |  |  |  |  | 38.664 |
|  |  |  |  |  |  | 23.785 |
|  |  |  |  |  |  | 39.463 |
|  |  |  |  | *Ercc1^-/D^* | 5 | 10.924 |
|  |  |  |  |  |  | 13.352 |
|  |  |  |  |  |  | 18.239 |
|  |  |  |  |  |  | 19.649 |
|  |  |  |  |  |  | 68.460 |
|  |  | 5 to 6 | *Puma* | WT | 5 | 0.839 |
|  |  |  |  |  |  | 1.274 |
|  |  |  |  |  |  | 0.861 |
|  |  |  |  |  |  | 0.801 |
|  |  |  |  |  |  | 1.356 |
|  |  |  |  | *Ckmm-Cre^+/-^;Ercc1^-/fl^* | 5 | 1.645 |
|  |  |  |  |  |  | 2.296 |
|  |  |  |  |  |  | 3.433 |
|  |  |  |  |  |  | 2.074 |
|  |  |  |  |  |  | 1.904 |
|  |  |  |  | *Ercc1^-/D^* | 5 | 2.947 |
|  |  |  |  |  |  | 2.323 |
|  |  |  |  |  |  | 1.519 |
|  |  |  |  |  |  | 1.948 |
|  |  |  |  |  |  | 2.381 |
|  |  | 5 to 6 | *Noxa* | WT | 5 | 0.769 |
|  |  |  |  |  |  | 1.198 |
|  |  |  |  |  |  | 1.466 |
|  |  |  |  |  |  | 1.096 |
|  |  |  |  |  |  | 0.675 |
|  |  |  |  | *Ckmm-Cre^+/-^;Ercc1^-/fl^* | 5 | 2.157 |
|  |  |  |  |  |  | 1.850 |
|  |  |  |  |  |  | 1.785 |
|  |  |  |  |  |  | 1.850 |
|  |  |  |  |  |  | 1.416 |
|  |  |  |  | *Ercc1^-/D^* | 5 | 1.778 |
|  |  |  |  |  |  | 1.267 |
|  |  |  |  |  |  | 2.357 |
|  |  |  |  |  |  | 1.840 |
|  |  |  |  |  |  | 1.265 |
|  |  | 5 to 6 | *Apaf1* | WT | 5 | 1.257 |
|  |  |  |  |  |  | 0.899 |
|  |  |  |  |  |  | 0.891 |
|  |  |  |  |  |  | 0.996 |
|  |  |  |  |  |  | 0.997 |
|  |  |  |  | *Ckmm-Cre^+/-^;Ercc1^-/fl^* | 5 | 1.771 |
|  |  |  |  |  |  | 1.346 |
|  |  |  |  |  |  | 2.135 |
|  |  |  |  |  |  | 1.519 |
|  |  |  |  |  |  | 1.292 |
|  |  |  |  | *Ercc1^-/D^* | 5 | 2.179 |
|  |  |  |  |  |  | 2.171 |
|  |  |  |  |  |  | 2.130 |
|  |  |  |  |  |  | 2.645 |
|  |  |  |  |  |  | 4.026 |
| Main figure 4e-j | Heart | 5 to 6 | *Bax* | *p53^+/-^;Ercc1^-/D^* | 5 | 7.245 |
|  |  |  |  |  |  | 8.519 |
|  |  |  |  |  |  | 4.605 |
|  |  |  |  |  |  | 2.369 |
|  |  |  |  |  |  | 3.699 |
|  |  | 5 to 6 | *Puma* | *p53^+/-^;Ercc1^-/D^* | 5 | 1.345 |
|  |  |  |  |  |  | 1.336 |
|  |  |  |  |  |  | 0.943 |
|  |  |  |  |  |  | 0.641 |
|  |  |  |  |  |  | 0.579 |
|  |  | 5 to 6 | *Noxa* | *p53^+/-^;Ercc1^-/D^* | 5 | 1.101 |
|  |  |  |  |  |  | 1.281 |
|  |  |  |  |  |  | 1.625 |
|  |  |  |  |  |  | 1.634 |
|  |  |  |  |  |  | 1.279 |
|  |  | 5 to 6 | *Apaf1* | *p53^+/-^;Ercc1^-/D^* | 5 | 1.081 |
|  |  |  |  |  |  | 0.615 |
|  |  |  |  |  |  | 0.572 |
|  |  |  |  |  |  | 0.423 |
|  |  |  |  |  |  | 0.172 |
|  |  | 5 to 6 | *Anp* | *p53^+/-^;Ercc1^-/D^* | 5 | 2.202 |
|  |  |  |  |  |  | 2.147 |
|  |  |  |  |  |  | 1.482 |
|  |  |  |  |  |  | 1.331 |
|  |  |  |  |  |  | 0.536 |
|  |  | 5 to 6 | *Bnp* | *p53^+/-^;Ercc1^-/D^* | 5 | 1.178 |
|  |  |  |  |  |  | 1.141 |
|  |  |  |  |  |  | 1.214 |
|  |  |  |  |  |  | 0.971 |
|  |  |  |  |  |  | 0.891 |
| Main figure 5d-h | Heart | 2 to 3 | *Cat* | WT | 5 | 0.639 |
|  |  |  |  |  |  | 1.403 |
|  |  |  |  |  |  | 1.337 |
|  |  |  |  |  |  | 0.638 |
|  |  |  |  |  |  | 1.310 |
|  |  |  |  | *Ckmm-Cre^+/-^;Ercc1^-/fl^* | 5 | 3.151 |
|  |  |  |  |  |  | 3.572 |
|  |  |  |  |  |  | 3.392 |
|  |  |  |  |  |  | 2.800 |
|  |  |  |  |  |  | 2.930 |
|  |  |  |  | *Ercc1^-/D^* | 5 | 3.031 |
|  |  |  |  |  |  | 1.766 |
|  |  |  |  |  |  | 4.305 |
|  |  |  |  |  |  | 2.222 |
|  |  |  |  |  |  | 1.637 |
|  |  | 5 to 6 |  | WT | 5 | 5.139 |
|  |  |  |  |  |  | 2.681 |
|  |  |  |  |  |  | 4.913 |
|  |  |  |  |  |  | 2.920 |
|  |  |  |  |  |  | 2.579 |
|  |  |  |  | *Ckmm-Cre^+/-^;Ercc1^-/fl^* | 5 | 2.878 |
|  |  |  |  |  |  | 8.089 |
|  |  |  |  |  |  | 6.273 |
|  |  |  |  |  |  | 9.410 |
|  |  |  |  |  |  | 5.643 |
|  |  |  |  | *Ercc1^-/D^* | 5 | 4.398 |
|  |  |  |  |  |  | 5.501 |
|  |  |  |  |  |  | 6.027 |
|  |  |  |  |  |  | 8.024 |
|  |  |  |  |  |  | 12.679 |
|  |  | 2 to 3 | *Hmox* | WT | 5 | 1.033 |
|  |  |  |  |  |  | 0.757 |
|  |  |  |  |  |  | 1.709 |
|  |  |  |  |  |  | 0.916 |
|  |  |  |  |  |  | 0.817 |
|  |  |  |  | *Ckmm-Cre^+/-^;Ercc1^-/fl^* | 5 | 6.971 |
|  |  |  |  |  |  | 3.312 |
|  |  |  |  |  |  | 2.117 |
|  |  |  |  |  |  | 1.927 |
|  |  |  |  |  |  | 1.779 |
|  |  |  |  | *Ercc1^-/D^* | 5 | 1.857 |
|  |  |  |  |  |  | 2.015 |
|  |  |  |  |  |  | 1.274 |
|  |  |  |  |  |  | 1.852 |
|  |  |  |  |  |  | 3.270 |
|  |  | 5 to 6 |  | WT | 5 | 24.712 |
|  |  |  |  |  |  | 35.777 |
|  |  |  |  |  |  | 36.954 |
|  |  |  |  |  |  | 25.201 |
|  |  |  |  |  |  | 59.650 |
|  |  |  |  | *Ckmm-Cre^+/-^;Ercc1^-/fl^* | 5 | 30.537 |
|  |  |  |  |  |  | 24.430 |
|  |  |  |  |  |  | 19.905 |
|  |  |  |  |  |  | 26.701 |
|  |  |  |  |  |  | 32.063 |
|  |  |  |  | *Ercc1^-/D^* | 5 | 59.445 |
|  |  |  |  |  |  | 79.395 |
|  |  |  |  |  |  | 34.150 |
|  |  |  |  |  |  | 31.820 |
|  |  |  |  |  |  | 128.549 |
|  |  | 2 to 3 | *Nqo1* | WT | 5 | 1.554 |
|  |  |  |  |  |  | 1.149 |
|  |  |  |  |  |  | 0.856 |
|  |  |  |  |  |  | 0.888 |
|  |  |  |  |  |  | 0.737 |
|  |  |  |  | *Ckmm-Cre^+/-^;Ercc1^-/fl^* | 5 | 4.734 |
|  |  |  |  |  |  | 12.035 |
|  |  |  |  |  |  | 9.454 |
|  |  |  |  |  |  | 11.790 |
|  |  |  |  |  |  | 8.987 |
|  |  |  |  | *Ercc1^-/D^* | 5 | 0.295 |
|  |  |  |  |  |  | 1.697 |
|  |  |  |  |  |  | 2.142 |
|  |  |  |  |  |  | 4.148 |
|  |  |  |  |  |  | 11.836 |
|  |  | 5 to 6 |  | WT | 5 | 14.391 |
|  |  |  |  |  |  | 25.133 |
|  |  |  |  |  |  | 13.800 |
|  |  |  |  |  |  | 20.742 |
|  |  |  |  |  |  | 19.193 |
|  |  |  |  | *Ckmm-Cre^+/-^;Ercc1^-/fl^* | 5 | 7.642 |
|  |  |  |  |  |  | 10.200 |
|  |  |  |  |  |  | 17.478 |
|  |  |  |  |  |  | 12.402 |
|  |  |  |  |  |  | 7.874 |
|  |  |  |  | *Ercc1^-/D^* | 5 | 8.664 |
|  |  |  |  |  |  | 19.477 |
|  |  |  |  |  |  | 11.351 |
|  |  |  |  |  |  | 6.384 |
|  |  |  |  |  |  | 20.637 |
|  | Heart LV | 5 to 6 | *Anp* | Control | 3 | 1.050 |
|  |  |  |  |  |  | 1.160 |
|  |  |  |  |  |  | 0.790 |
|  |  |  |  | *Ckmm-Cre^+/-^;Ercc1^-/fl^* | 3 | 3.680 |
|  |  |  |  |  |  | 3.100 |
|  |  |  |  |  |  | 4.350 |
|  |  |  |  | mitCAT-*Ckmm-Cre^+/-^;Ercc1^-/fl^* | 3 | 1.450 |
|  |  |  |  |  |  | 1.850 |
|  |  |  |  |  |  | 2.340 |
|  |  |  | *Bnp* | Control | 3 | 1.090 |
|  |  |  |  |  |  | 0.920 |
|  |  |  |  |  |  | 0.990 |
|  |  |  |  | *Ckmm-Cre^+/-^;Ercc1^-/fl^* | 3 | 2.490 |
|  |  |  |  |  |  | 1.980 |
|  |  |  |  |  |  | 3.270 |
|  |  |  |  | mitCAT-*Ckmm-Cre^+/-^;Ercc1^-/fl^* | 3 | 1.230 |
|  |  |  |  |  |  | 1.010 |
|  |  |  |  |  |  | 1.450 |
| Supplementary Figure 2a (accompanied with main figure 1a data) | Heart | 2 to 3 | *Ercc1* | *Ercc1^-/D^* | 5 | 0.011 |
|  |  |  |  |  |  | 0.006 |
|  |  |  |  |  |  | 0.015 |
|  |  |  |  |  |  | 0.007 |
|  |  |  |  |  |  | 0.084 |
|  | Skeletal muscle |  |  | *Ercc1^-/D^* | 4 | 0.051 |
|  |  |  |  |  |  | 0.168 |
|  |  |  |  |  |  | 0.032 |
|  |  |  |  |  |  | 0.104 |
|  | Liver |  |  | *Ercc1^-/D^* | 3 | 0.044 |
|  |  |  |  |  |  | 0.024 |
|  |  |  |  |  |  | 0.053 |
|  | Kidney |  |  | *Ercc1^-/D^* | 5 | 0.030 |
|  |  |  |  |  |  | 0.081 |
|  |  |  |  |  |  | 0.004 |
|  |  |  |  |  |  | 0.001 |
|  |  |  |  |  |  | 0.001 |
| Supplementary Figure 11a-d | Heart LV | 2 to 3 | *Col1a1* | WT | 5 | 1.511 |
|  |  |  |  |  |  | 1.043 |
|  |  |  |  |  |  | 0.734 |
|  |  |  |  |  |  | 0.662 |
|  |  |  |  |  |  | 1.306 |
|  |  |  |  | *Ckmm-Cre^+/-^;Ercc1^-/fl^* | 5 | 0.596 |
|  |  |  |  |  |  | 0.551 |
|  |  |  |  |  |  | 0.419 |
|  |  |  |  |  |  | 0.599 |
|  |  |  |  |  |  | 1.122 |
|  |  | 2 to 3 | *Col3a* | WT | 5 | 1.502 |
|  |  |  |  |  |  | 1.183 |
|  |  |  |  |  |  | 0.857 |
|  |  |  |  |  |  | 0.845 |
|  |  |  |  |  |  | 0.777 |
|  |  |  |  | *Ckmm-Cre^+/-^;Ercc1^-/fl^* | 5 | 0.358 |
|  |  |  |  |  |  | 0.388 |
|  |  |  |  |  |  | 0.805 |
|  |  |  |  |  |  | 0.764 |
|  |  |  |  |  |  | 1.381 |
|  |  | 2 to 3 | *Anp* | WT | 5 | 1.547 |
|  |  |  |  |  |  | 0.814 |
|  |  |  |  |  |  | 0.963 |
|  |  |  |  |  |  | 0.770 |
|  |  |  |  |  |  | 1.071 |
|  |  |  |  | *Ckmm-Cre^+/-^;Ercc1^-/fl^* | 5 | 0.489 |
|  |  |  |  |  |  | 0.109 |
|  |  |  |  |  |  | 0.272 |
|  |  |  |  |  |  | 1.029 |
|  |  |  |  |  |  | 0.320 |
|  |  | 2 to 3 | *Bnp* | WT | 5 | 0.897 |
|  |  |  |  |  |  | 0.713 |
|  |  |  |  |  |  | 0.820 |
|  |  |  |  |  |  | 1.074 |
|  |  |  |  |  |  | 1.778 |
|  |  |  |  | *Ckmm-Cre^+/-^;Ercc1^-/fl^* | 5 | 0.242 |
|  |  |  |  |  |  | 0.177 |
|  |  |  |  |  |  | 0.213 |
|  |  |  |  |  |  | 1.125 |
|  |  |  |  |  |  | 0.593 |
| Supplementary Figure 13a-d | Heart | 2 to 3 | *Bax* | WT | 5 | 0.765 |
|  |  |  |  |  |  | 1.192 |
|  |  |  |  |  |  | 1.138 |
|  |  |  |  |  |  | 0.939 |
|  |  |  |  |  |  | 1.026 |
|  |  |  |  | *Ckmm-Cre^+/-^;Ercc1^-/fl^* | 5 | 2.277 |
|  |  |  |  |  |  | 2.488 |
|  |  |  |  |  |  | 1.829 |
|  |  |  |  |  |  | 3.374 |
|  |  |  |  |  |  | 6.081 |
|  |  |  |  | *Ercc1^-/D^* | 5 | 6.371 |
|  |  |  |  |  |  | 2.191 |
|  |  |  |  |  |  | 2.246 |
|  |  |  |  |  |  | 1.670 |
|  |  |  |  |  |  | 4.349 |
|  |  | 2 to 3 | *Puma* | WT | 5 | 0.615 |
|  |  |  |  |  |  | 1.390 |
|  |  |  |  |  |  | 0.916 |
|  |  |  |  |  |  | 1.308 |
|  |  |  |  |  |  | 0.977 |
|  |  |  |  | *Ckmm-Cre^+/-^;Ercc1^-/fl^* | 5 | 2.339 |
|  |  |  |  |  |  | 2.473 |
|  |  |  |  |  |  | 3.453 |
|  |  |  |  |  |  | 1.047 |
|  |  |  |  |  |  | 2.093 |
|  |  |  |  | *Ercc1^-/D^* | 5 | 1.808 |
|  |  |  |  |  |  | 2.113 |
|  |  |  |  |  |  | 1.515 |
|  |  |  |  |  |  | 1.518 |
|  |  |  |  |  |  | 3.978 |
|  |  | 2 to 3 | *Noxa* | WT | 5 | 0.709 |
|  |  |  |  |  |  | 0.903 |
|  |  |  |  |  |  | 0.930 |
|  |  |  |  |  |  | 1.485 |
|  |  |  |  |  |  | 1.132 |
|  |  |  |  | *Ckmm-Cre^+/-^;Ercc1^-/fl^* | 5 | 1.741 |
|  |  |  |  |  |  | 1.847 |
|  |  |  |  |  |  | 2.286 |
|  |  |  |  |  |  | 1.992 |
|  |  |  |  |  |  | 1.953 |
|  |  |  |  | *Ercc1^-/D^* | 5 | 3.445 |
|  |  |  |  |  |  | 2.506 |
|  |  |  |  |  |  | 1.830 |
|  |  |  |  |  |  | 2.013 |
|  |  |  |  |  |  | 3.806 |
|  |  | 2 to 3 | *Apaf1* | WT | 5 | 0.764 |
|  |  |  |  |  |  | 0.675 |
|  |  |  |  |  |  | 1.457 |
|  |  |  |  |  |  | 1.201 |
|  |  |  |  |  |  | 1.108 |
|  |  |  |  | *Ckmm-Cre^+/-^;Ercc1^-/fl^* | 4 | 3.719 |
|  |  |  |  |  |  | 4.908 |
|  |  |  |  |  |  | 3.120 |
|  |  |  |  |  |  | 2.820 |
|  |  |  |  | *Ercc1^-/D^* | 5 | 2.704 |
|  |  |  |  |  |  | 1.997 |
|  |  |  |  |  |  | 1.956 |
|  |  |  |  |  |  | 2.150 |
|  |  |  |  |  |  | 2.181 |
